# *Lactobacillus rhamnosus* GG and *Lactobacillus paracasei* IMPC2.1 Mitigate LPS-Induced Epithelial Barrier Dysfunction: A Focus on Autophagy Regulation

**DOI:** 10.3390/ijms262211148

**Published:** 2025-11-18

**Authors:** Antonella Orlando, Fatima Maqoud, Domenica Mallardi, Simona Drago, Eleonora Malerba, Guglielmina Chimienti, Francesco Russo

**Affiliations:** 1 Functional Gastrointestinal Disorders Research Group, National Institute of Gastroenterology IRCCS “S. de Bellis”, 70013 Castellana Grotte, Italy; antonella.orlando@irccsdebellis.it (A.O.); fatima.maqoud@irccsdebellis.it (F.M.); domenica.mallardi@irccsdebellis.it (D.M.); simona.drago@irccsdebellis.it (S.D.); eleonora.malerba@irccsdebellis.it (E.M.); 2 Department of Biosciences, Biotechnology and Environment, University di Bari “Aldo Moro”, 70100 Bari, Italy; guglielminaalessandra.chimienti@uniba.it

**Keywords:** probiotics, *Lactobacillus rhamnosus GG*, *Lactobacillus paracasei*, lipopolysaccharide (LPS), intestinal permeability, autophagy, inflammation

## Abstract

The intestinal epithelial barrier is critical for maintaining gut homeostasis, yet its integrity can be compromised by inflammation and microbial dysbiosis. Here, we demonstrate that *Lactobacillus rhamnosus GG* (LGG) and *Lactobacillus paracasei* IMPC2.1 (*L. paracasei*) show their effectiveness in enhancing epithelial barrier function and modulating autophagy, counteract the epithelial barrier dysfunction, induced by Lipopolysaccharide (LPS), in Caco-2 cells by modulating tight junction (TJ) protein expression through regulation of inflammation and apoptosis. LPS exposure significantly reduced transepithelial electrical resistance (TEER) and increased paracellular permeability, effects that were partially reversed by both probiotic strains. Western blot analysis revealed that LPS downregulated ZO-1, Occludin, and p-mTOR, while upregulating autophagy markers LC3-II and Beclin1, without affecting p62 levels. The latter finding indicated an impairment of autophagy flux, confirmed by immunofluorescence experiments. Co-treatment with LGG or *L. paracasei* restored TJ protein expression and alleviated the LPS-induced impairment of autophagic flux. Both probiotics suppressed LPS-induced cyclooxygenase-2 (Cox-2) and Bax upregulation, suggesting anti-inflammatory and anti-apoptotic effects. In the complex interplay between inflammation, autophagy, and apoptosis, these findings highlight a key regulatory mechanism in probiotic-mediated epithelial protection, underscoring the therapeutic potential of LGG and *L. paracasei* in mitigating gut barrier dysfunction.

## 1. Introduction

The intestinal epithelium, made up of a single layer of columnar epithelial cells, acts as a dynamic and selective barrier that separates the gut lumen from the body’s internal environment. In addition to its role in nutrient and water absorption, this barrier is the body’s first line of defense against pathogens and harmful antigens [1].

At the core of barrier integrity are tight junctions (TJs), multiprotein complexes that seal the space between neighboring epithelial cells. Located at the apical-lateral membrane, TJs are primarily formed from transmembrane proteins like claudins and occludin, supported by intracellular scaffolding proteins such as ZO-1, ZO-2, and ZO-3. These intracellular components anchor the TJs to the actin cytoskeleton, providing both structure and stability [2,3]. Disruption of TJs weakens the epithelial barrier and has been associated with various pathological conditions.

Among the strategies to preserve barrier function, probiotics have gained considerable interest. Usually taken as dietary supplements, probiotics (especially *Lactobacillus* species) enhance gut health through various mechanisms, including immune modulation and strengthening of TJ protein expression and localization [4,5]. These bacteria can also counteract epithelial damage caused by inflammatory mediators or pathogenic toxins by activating innate immune receptors such as Toll-like receptors (TLRs) [6].

Lipopolysaccharide (LPS), a primary component of the outer membrane of Gram-negative bacteria, is a key ligand for TLR4. When LPS enters the circulation—often due to gut dysbiosis—it triggers strong immune and inflammatory responses [7,8,9]. In a dysbiotic gut, microbes that produce LPS proliferate, leading to increased LPS production and translocation across a weakened intestinal barrier. This process activates the TLR4 signaling pathway, amplifying both local and systemic inflammation and further compromising barrier integrity, a cycle often referred to as “leaky gut” [10].

TLR4, as a transmembrane receptor, plays a central role in pathogen recognition and immune activation. Upon LPS binding, TLR4 recruits adaptor proteins, notably Myeloid Differentiation Primary Response Gene 88 (MyD88), which triggers downstream signaling through Tumor Necrosis Factor Receptor-Associated Factor 6 (TRAF6). This signaling activates either the mitogen-activated protein kinase (MAPK) pathways or the inhibitor of κB kinase (IKK) complex, ultimately leading to the activation of nuclear factor κB (NF-κB) and the transcription of inflammatory genes, including *IL-1β*, *IL-6*, *IL-8*, *iNOS*, *COX-2*, and *PGE2* [11].

LPS is also known to induce autophagy in intestinal epithelial cells [12]. Autophagy is a carefully controlled, evolutionarily conserved process that breaks down damaged organelles and misfolded proteins through lysosomal degradation [13]. Under normal conditions, basal autophagy helps protect and maintain gut health and microbial defense [14]. However, during stress or disease, autophagic responses can become unbalanced [15]. In the gut, faulty autophagy has been linked to the development of inflammatory bowel disease (IBD) and might contribute to epithelial dysfunction [16]. While autophagy can be a protective response, excessive activation may cause cell damage or death, highlighting its dual role.

Several molecular markers are used to monitor autophagy, including microtubule-associated protein 1 light chain (LC3), Beclin1, and p62 [17,18]. Autophagy is regulated by multiple signaling pathways, especially the AKT/mTOR axis, a negative regulator of autophagy, and the MAPK pathways (ERK1/2, JNK, p38), which influence gene expression associated with autophagic activity [19,20].

The connection between dysbiosis-induced LPS translocation and ongoing TLR4 activation underscores a crucial therapeutic target in diseases caused by chronic low-grade inflammation. Restoring gut barrier function, by adjusting the microbiota or strengthening epithelial integrity, offers hope for breaking the inflammation cycle and maintaining intestinal balance. Intestinal dysbiosis, often characterized by increased production of lipopolysaccharide (LPS) and loss of epithelial integrity, is emerging as a central mediator linking gut microbial imbalance to systemic disease. Beyond direct microbial modulation, dietary components profoundly shape the gut environment and influence disease outcomes: for example, phenolic compounds exert prebiotic effects by selectively promoting the growth of beneficial bacteria, including *Lactobacillus* and *Bifidobacterium* species, thereby enhancing short-chain fatty acid production, epithelial repair, and immune tolerance. Similarly, the quality of dietary fats modulates inflammation and metabolic risk, as evidenced by the divergent effects of cocoa butter versus n-3-rich fish oil on obesity-associated tumor progression [21]. In parallel, recent evidence highlights that metabolite produced by probiotics, including major microbial fermentation products, can directly modulate intestinal barrier function and local immune responses. While most studies have focused on isolated microbial products, the role of live bacteria in orchestrating host–microbe interactions remain incompletely understood [22,23,24].

Growing evidence suggests that autophagy is a key pathway through which probiotics provide their protective effects [25]. Some studies indicate that probiotics can influence autophagy-related signaling, supporting epithelial homeostasis, preserving mucus layer function, and reducing inflammation [26,27,28]. However, despite increasing interest, the exact mechanisms by which probiotics regulate autophagy, especially in the context of LPS-induced epithelial dysfunction, remain poorly understood.

Autophagy is a double-edged sword in epithelial cells, as it can either protect them from stress or worsen injury when dysregulated [13,16]. While the role of probiotics in maintaining TJ integrity and reducing inflammation has been well-documented, their mechanism of modulating autophagy under inflammatory conditions remains less understood. This knowledge gap limits our ability to fully harness their therapeutic potential, especially in disorders like irritable bowel syndrome (IBS) or metabolic diseases with gut barrier dysfunction.

Our previous studies have shown that *Lactobacillus rhamnosus* GG (LGG) can restore TJ integrity and decrease paracellular permeability after gliadin exposure, both in vitro and in vivo, by altering the expression and placement of key TJ proteins [29,30]. Similarly, *Lactobacillus paracasei* IMPC2.1 (*L. paracasei*), a strain with proven ability to colonize, has demonstrated beneficial effects on gut microbiota composition, including suppression of pathobionts like *E. coli* and *Clostridium* spp., as well as modest modulation of SCFA production and enzymatic activity [31]. In vitro data also emphasize its antiproliferative and pro-apoptotic properties in gastrointestinal cancer cells, indicating broader roles in mucosal health and disease prevention [32].

Despite these findings, the molecular mechanisms by which these probiotics protect the intestinal barrier under inflammatory conditions remain incompletely understood. While their ability to preserve TJ architecture and modulate immune responses is increasingly recognized [29,32], the interplay between probiotic action, autophagy, and barrier resilience, particularly in the context of LPS-induced injury, is still to be investigated.

Using the Caco-2 cell line, a well-established in vitro model that recapitulates the morphological and functional properties of human intestinal enterocytes, we evaluated the effects of LGG and *L. paracasei* on TJ protein expression, autophagic signaling, and inflammatory responses in an LPS-challenged model of epithelial injury [33].

Given the key role of autophagy in epithelial resilience and its reported disruption under LPS-driven inflammation, we hypothesize that LGG and *L. paracasei* counteract LPS-induced barrier dysfunction also through the regulation of autophagy.

However, most studies have investigated effects mediated by isolated microbial products, leaving the role of live bacteria in directly shaping host-microbe interactions still partially unexplored. This study aims to define the mechanistic interplay between probiotic intervention, autophagy modulation, and TJ stabilization, providing a more direct understanding of the mechanisms by which probiotics influence intestinal physiology, thus providing new insights into the therapeutic potential of these strains in conditions characterized by intestinal barrier failure.

## 2. Results

### 2.1. Caco-2 Cell Viability

**Figure 1:**
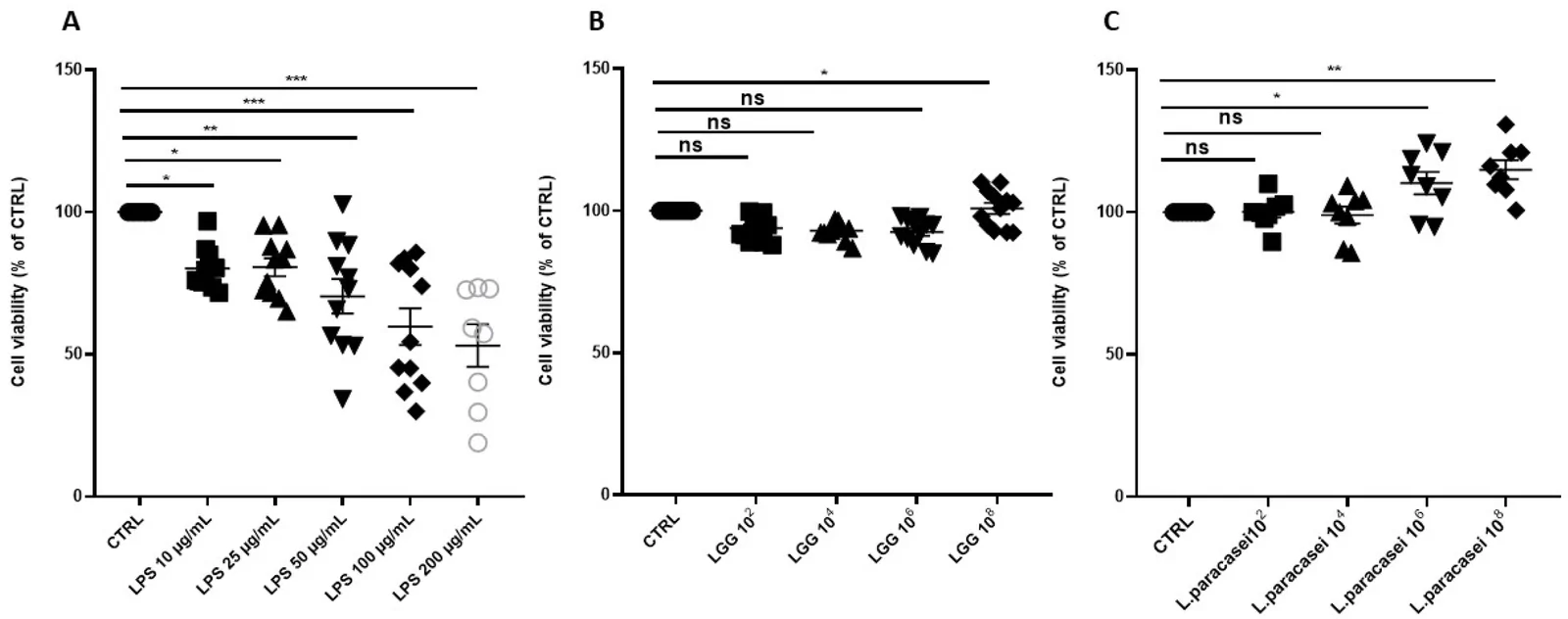
Percentage change of cell viability of Caco-2 monolayer after 24 h of treatment. (**A**) Caco-2 cells untreated (CTRL), treated with increasing concentrations of LPS (from 10 μg/mL to 200 μg/mL), for 24 h. (**B**) Caco-2 cells untreated (CTRL), treated with increasing concentrations of LGG (from 10^2^ CFU/mL to 10^8^ CFU/mL), for 24 h. (**C**) Caco-2 cells untreated (CTRL), treated with increasing concentrations of *L. paracasei* (from 10^2^ CFU/mL to 10^8^ CFU/mL), for 24 h. All data represent the result of at least three experiments (mean ± SEM). *ns* (not significant), * *p* < 0.05, ** *p* < 0.01, *** *p* < 0.001 compared to control group.

In a preliminary set of experiments, Caco-2 cells were exposed to LPS at concentrations ranging from 10 to 200 µg/mL for 24 h, and cell viability was assessed using the MTT assay. As shown in Figure 1A, LPS induced a dose-dependent reduction in cell viability.

Treatment with LGG or *L. paracasei* at concentrations ranging from 10^2^ to 10^8^ CFU/mL for 24 h did not significantly affect cell viability at concentrations up to 10^8^ CFU/mL for LGG and 10^6^ CFU/mL for *L. paracasei*. However, higher concentrations (10^8^ CFU/mL) of both Lactobacillus strains significantly increased cell viability (Figure 1B,C). The increase in viability at 10^8^ CFU/mL may reflect trophic effects of probiotic metabolites (e.g., vitamins, SCFAs) that support epithelial metabolism, as previously reported [24].

**Figure 2:**
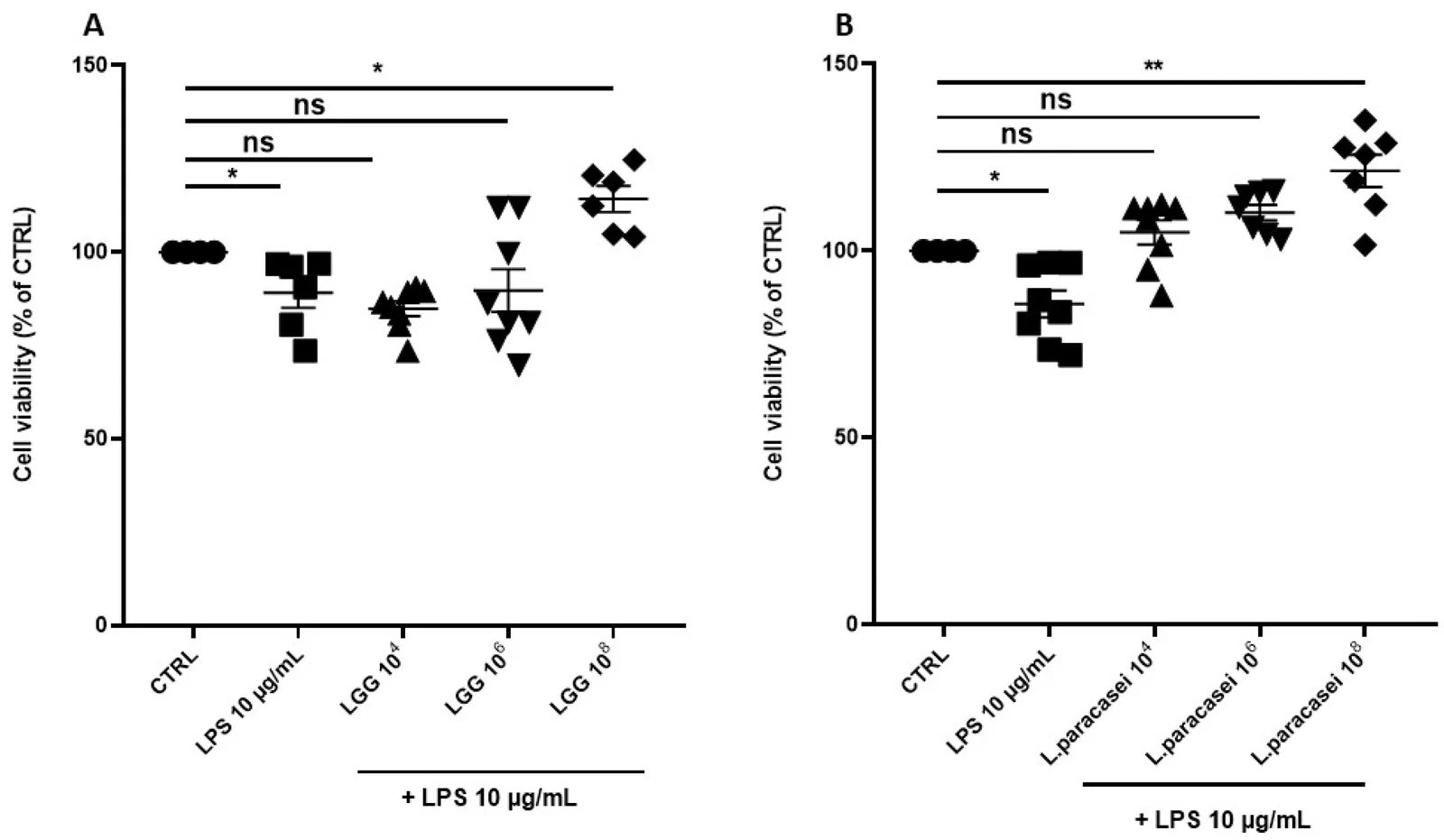
Percentage change of cell viability of Caco-2 monolayer after 24 h of treatment. (**A**) Caco-2 cells untreated (CTRL), treated with LPS 10 μg/mL, LPS 10 μg/mL + LGG 10^4^ CFU/mL, LPS 10 μg/mL + LGG 10^6^ CFU/mL, LPS 10 μg/mL + LGG 10^8^ CFU/mL, for 24 h. (**B**) Caco-2 cells untreated (CTRL), treated with LPS 10 μg/mL, LPS 10 μg/mL + *L. paracasei* 10^4^ CFU/mL, LPS 10 μg/mL + *L. paracasei* 10^6^ CFU/mL, LPS 10 μg/mL + *L. paracasei* 10^8^ CFU/mL, for 24 h. All data represent the result of at least three experiments (mean ± SEM). *ns* (not significant), * *p* < 0.05, ** *p* < 0.01, compared to control group.

To assess the protective effects of probiotics, Caco-2 cells were exposed to LPS (10 µg/mL), a dose not inducing mortality above 10%. LPS was incubated alone or in co-culture with different concentrations of LGG and *L. paracasei* (10^4^ to 10^8^ CFU/mL) for 24 h (Figure 2A,B). Cell viability, expressed as a percentage of the untreated control, remained above 95% in the groups co-treated with LGG or *L. paracasei* at 10^8^ CFU/mL. Based on these findings, a concentration of 10^8^ CFU/mL was chosen for subsequent experiments.

### 2.2. TEER Measurements

**Figure 3:**
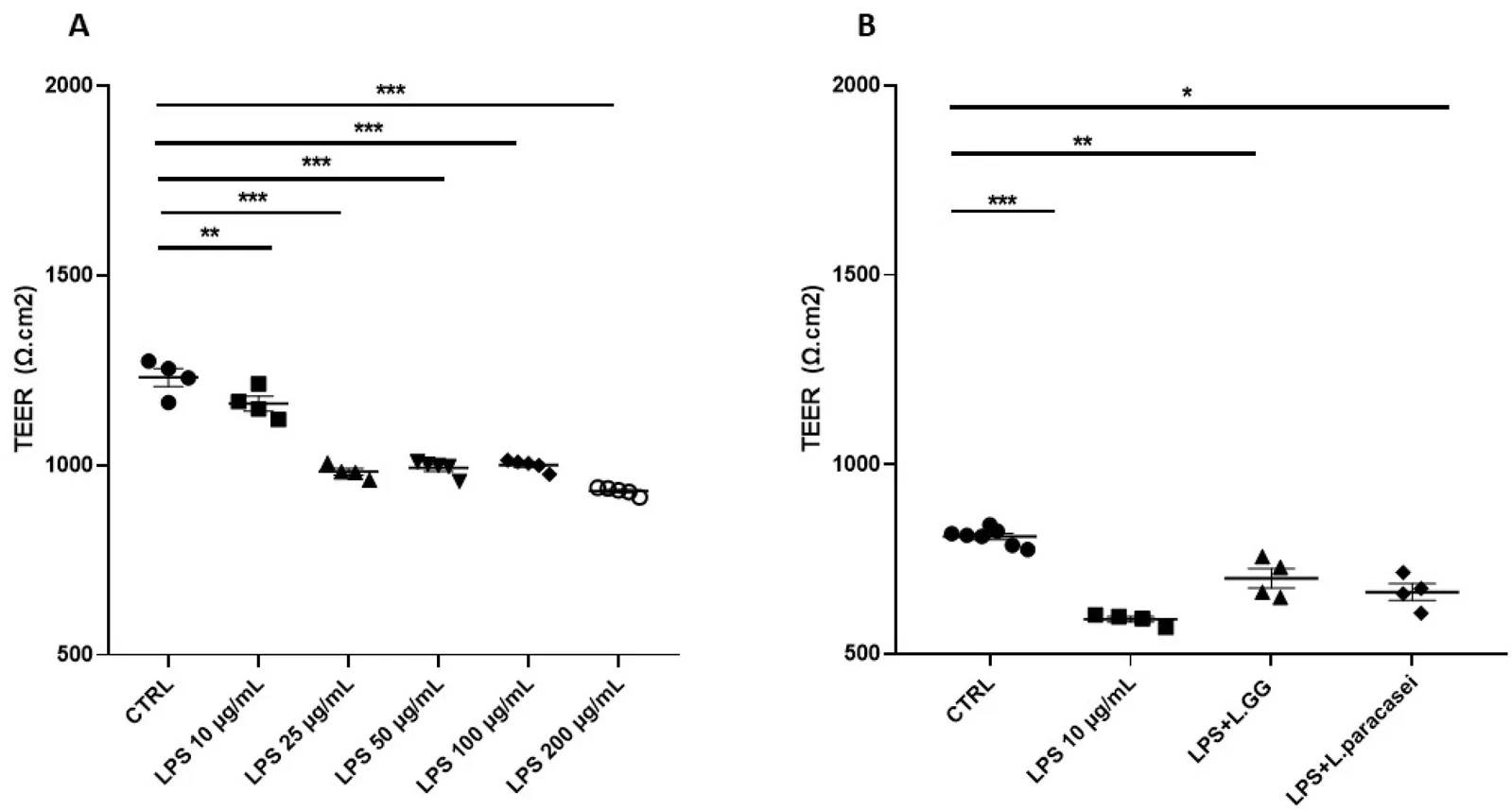
TEER measurement (expressed as Ω cm^2^) of Caco-2 monolayer after 24 h of treatment. (**A**) Caco-2 cells untreated (CTRL), treated with increasing concentrations of LPS (from 10 μg/mL to 200 μg/mL), for 24 h. (**B**) Caco-2 cells untreated (CTRL), treated with LPS 10 μg/mL, LPS 10 μg/mL + LGG 10^8^ CFU/mL (LPS + LGG), LPS 10 μg/mL + *L. paracasei* 10^8^ CFU/mL (LPS + *L. paracasei*), for 24 h. All data represent the result of at least three experiments (mean ± SEM). * *p* < 0.05, ** *p* < 0.01, *** *p* < 0.001 compared to control group.

To assess the impact of LPS on intestinal epithelial integrity, Caco-2 monolayers were cultured on transwell inserts for 15 days and then exposed to LPS at concentrations ranging from 10 to 200 µg/mL for 24 h. All LPS tested concentrations led to a significant decrease of TEER values compared to CTRL group (Figure 3A). In particular, treatment with LPS (10 µg/mL) significantly reduced TEER by 27.0% compared to the control (*p* < 0.001). Co-treatment with LGG or *L. paracasei* attenuated this effect, reducing TEER by 13.6% (*p* = 0.002) and 18.1% (*p* < 0.001), respectively, corresponding to recoveries of 13.4% and 8.9% compared to LPS alone. Consequently, LGG and *L. paracasei* increased TEER by 18.2% (*p* = 0.0012) and 14.7% (*p* = 0.048) compared to LPS, indicating a partial restoration of barrier integrity (Figure 3B).

### 2.3. Paracellular Permeability

**Figure 4:**
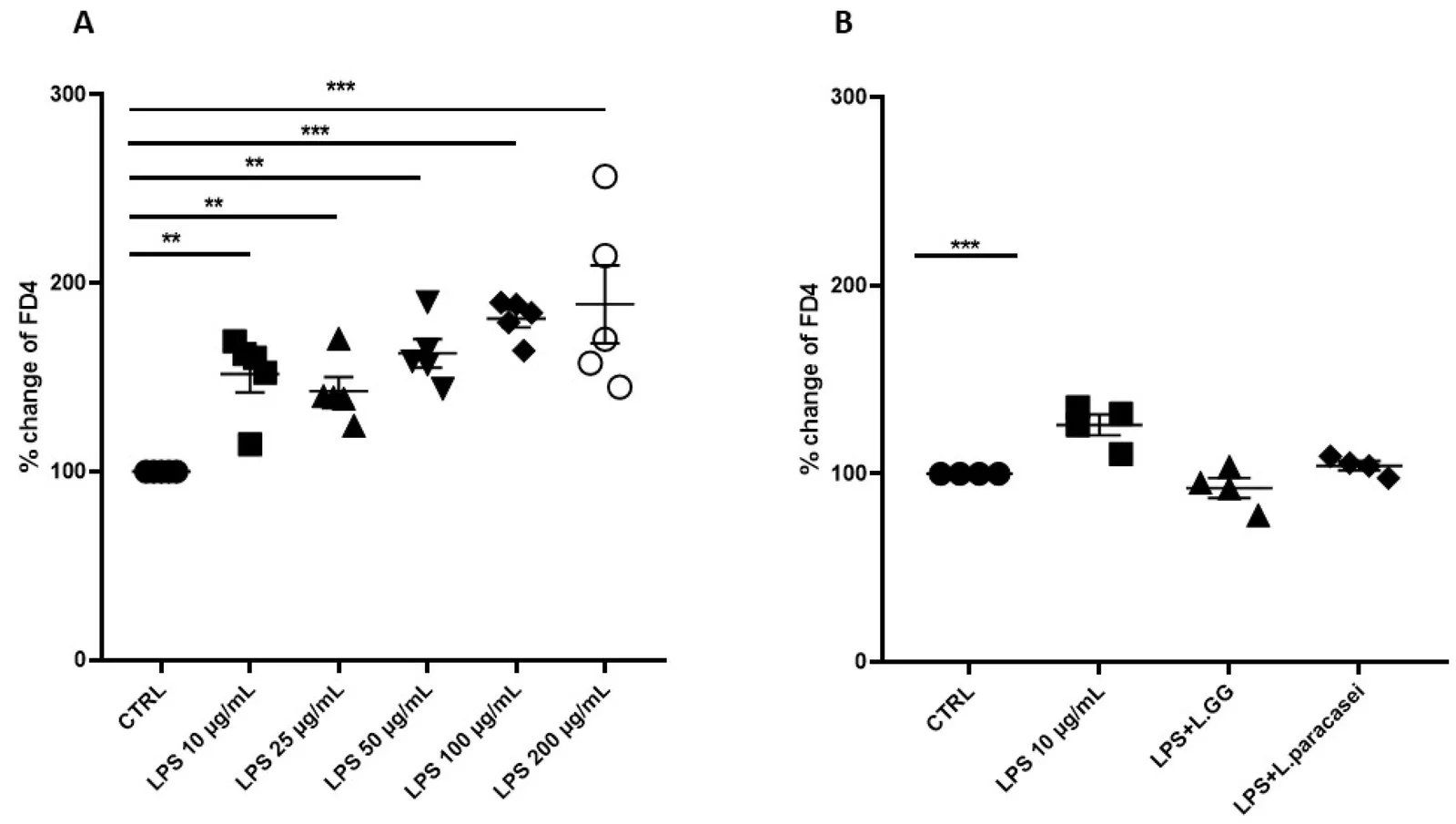
Percentage of change of FITC-dextran 4 (FD4) fluorescence intensity of Caco-2 monolayer after 24 h of treatment. (**A**) Caco-2 cells untreated (CTRL), treated with increasing concentrations of LPS (from 10 μg/mL to 200 μg/mL), for 24 h. (**B**) Caco-2 cells untreated (CTRL), treated with LPS 10 μg/mL, LPS 10 μg/mL + LGG 10^8^ CFU/mL (LPS + LGG), LPS 10 μg/mL + *L. paracasei* 10^8^ CFU/mL (LPS + *L. paracasei*), for 24 h. All data represent the result of at least three experiments (mean ± SEM). ** *p* < 0.01, *** *p* < 0.001 compared to control group.

To evaluate paracellular transport, the percentage change in FD4 fluorescence intensity in the basolateral compartment of the transwell system was measured. A differentiated Caco-2 cell monolayer was exposed to increasing concentrations of LPS (10 to 200 µg/mL) for 24 h (Figure 4A). The percentage change in FD4 fluorescence intensity significantly increased in all LPS-treated groups compared to untreated controls, indicating enhanced paracellular permeability.

To investigate the potential protective effects of LGG and *L. paracasei* against LPS-induced barrier dysfunction, Caco-2 monolayers were co-treated with LPS (10 µg/mL) and either LGG or *L. paracasei* at a concentration of 10^8^ CFU/mL. Co-administration of LPS with LGG or *L. paracasei* reduced FD4 fluorescence intensity compared to the LPS-only group, indicating a trend toward improved barrier integrity; however, this reduction was not statistically significant (*p* > 0.05) (Figure 4B).

The above showed results further support our choice to select an LPS concentration of 10 µg/mL for all subsequent experiments. Indeed, this dosage effectively induced intestinal epithelial barrier disruption while preserving acceptable cell viability. This finding is consistent with several studies in the literature [34,35,36].

Furthermore, the experimental conditions employed in this study are consistent with a state of low-grade inflammation, which may be relevant to IBS, a condition of particular interest to our research group. Conversely, exposure to 25 µg/mL LPS resulted in a marked decrease in cell viability to approximately 88%, thereby complicating the interpretation of barrier-related outcomes.

### 2.4. Tight Junction Expression

**Figure 5:**
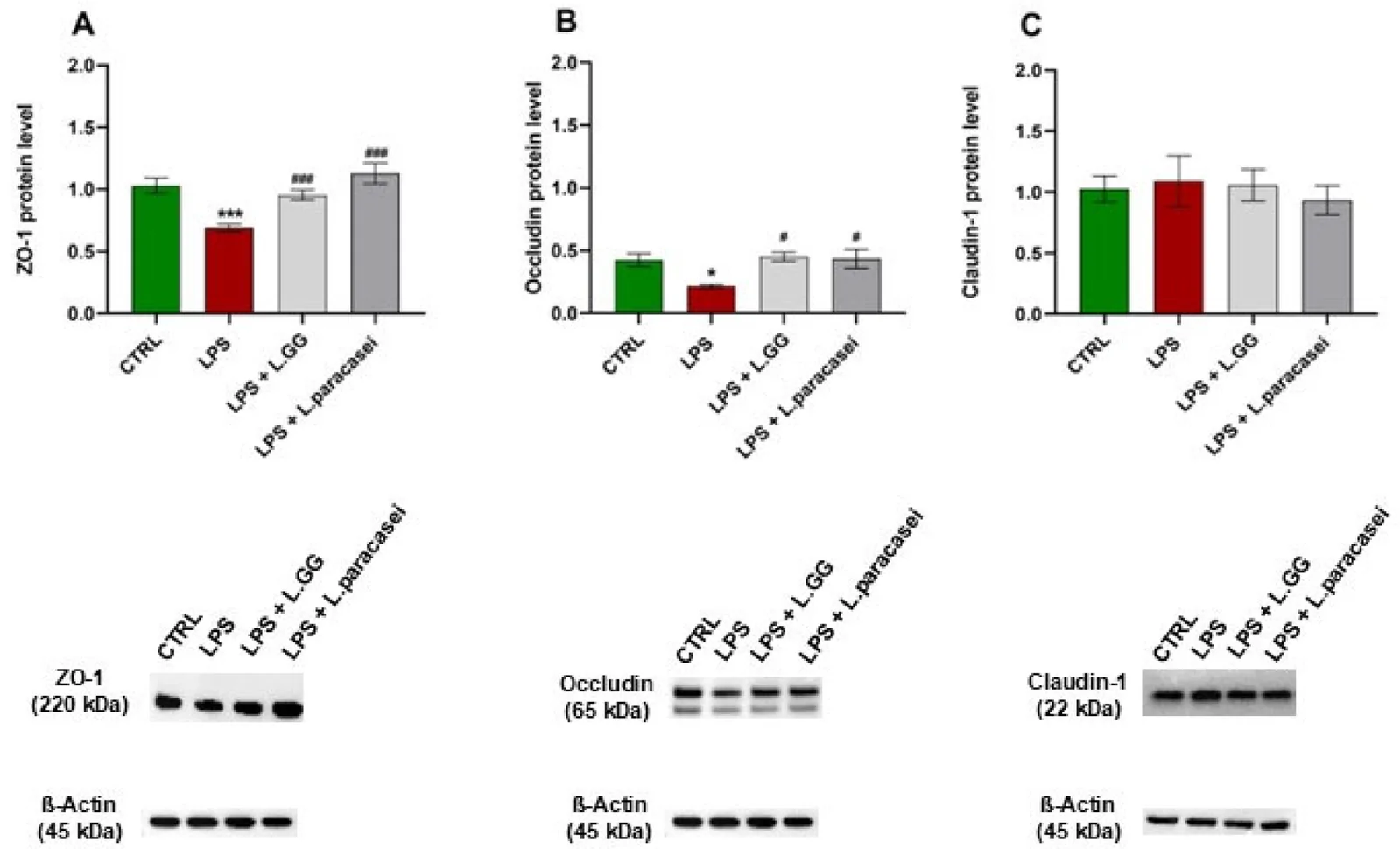
Western blot analysis of ZO-1 (**A**), Occludin (**B**) and Claudin-1 (**C**) proteins in Caco-2 cells, untreated (CTRL), treated with LPS 10 μg/mL (LPS), LPS 10 μg/mL + LGG 10^8^ CFU/mL (LPS + LGG), LPS 10 μg/mL + *L. paracasei* 10^8^ CFU/mL (LPS + *L. paracasei*), for 24 h. All data represent the result of at least three experiments (mean ± SEM). * *p* < 0.05, *** *p* < 0.001 compared to control group; ^#^ *p* < 0.05, ^###^ *p* < 0.001 compared to LPS group.

The expression of TJ proteins—ZO-1, Occludin, and Claudin-1—was analyzed using Western blot. In the LPS-treated group, ZO-1 and Occludin protein levels decreased significantly by 33.0% and 48.9%, respectively, compared to untreated controls (Figure 5A,B). There was no significant change in Claudin-1 protein levels (Figure 5C).

Co-administration of LPS with LGG significantly increased ZO-1 and Occludin protein levels by 38.4% and 108.3%, respectively, compared to the LPS-only group, counteracting the LPS-induced reduction. Similarly, co-administration of LPS with *L. paracasei* increased ZO-1 and Occludin levels by 63.6% and 101.2%, respectively, compared to the LPS-treated group. No significant effect on Claudin-1 protein levels was observed in any of the treatment groups.

### 2.5. Signal Pathways Regulating Autophagy

**Figure 6:**
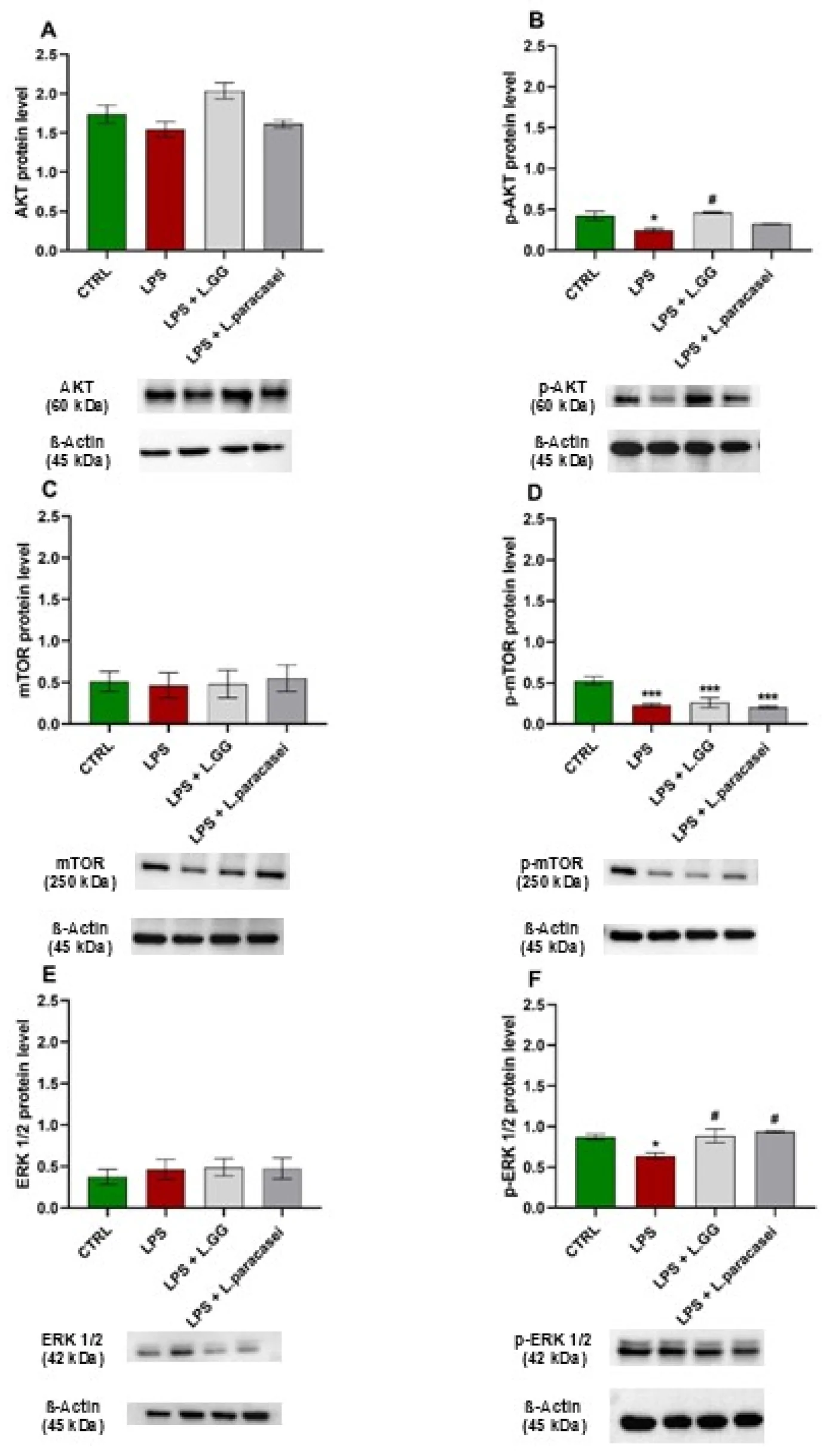
Western blot analysis of AKT (**A**), p-AKT (**B**), mTOR (**C**), p-mTOR (**D**), ERK 1/2 (**E**), and p-ERK 1/2 (**F**) proteins in Caco-2 cells untreated (CTRL), treated with LPS 10 μg/mL (LPS), LPS 10 μg/mL + LGG 10^8^ CFU/mL (LPS + LGG), LPS 10 μg/mL + *L. paracasei* 10^8^ CFU/mL (LPS + *L. paracasei*), for 24 h. All data represent the result of at least three experiments (mean ± SEM). * *p* < 0.05, *** *p* < 0.001 compared to the control group. ^#^ *p* < 0.05 compared to LPS group.

To examine the roles of LPS and Lactobacilli in signaling pathways involved in autophagy regulation, specifically the AKT/mTOR and ERK pathways, the protein levels of AKT, p-AKT, mTOR, p-mTOR, ERK1/2, and p-ERK1/2 were measured. Exposure of Caco-2 cells to LPS for 24 h significantly changed the protein levels of p-AKT, p-mTOR, and p-ERK 1/2, with decreases of −41.2%, −56.3%, and −26.7%, respectively, compared to untreated controls (Figure 6B,D,F).

Co-administration of LPS with LGG significantly counteracted the LPS-induced reduction in p-AKT levels (by 86.4%). The same effect was observed when LPS was co-treated with *L. paracasei*, although this increase did not reach statistical significance. Co-incubation with LPS + LGG resulted in less reduction of p-mTOR levels (−50.8%), while co-incubation with LPS + *L. paracasei* caused a 61.3% reduction compared to control cells (Figure 6D).

Regarding p-ERK 1/2 levels, the co-incubation of LPS + LGG and LPS + *L. paracasei* resulted in a significant enhancement by 38.8% and 46.8%, respectively, in comparison with LPS-treated cells (Figure 6F).

### 2.6. Autophagy Markers

**Figure 7:**
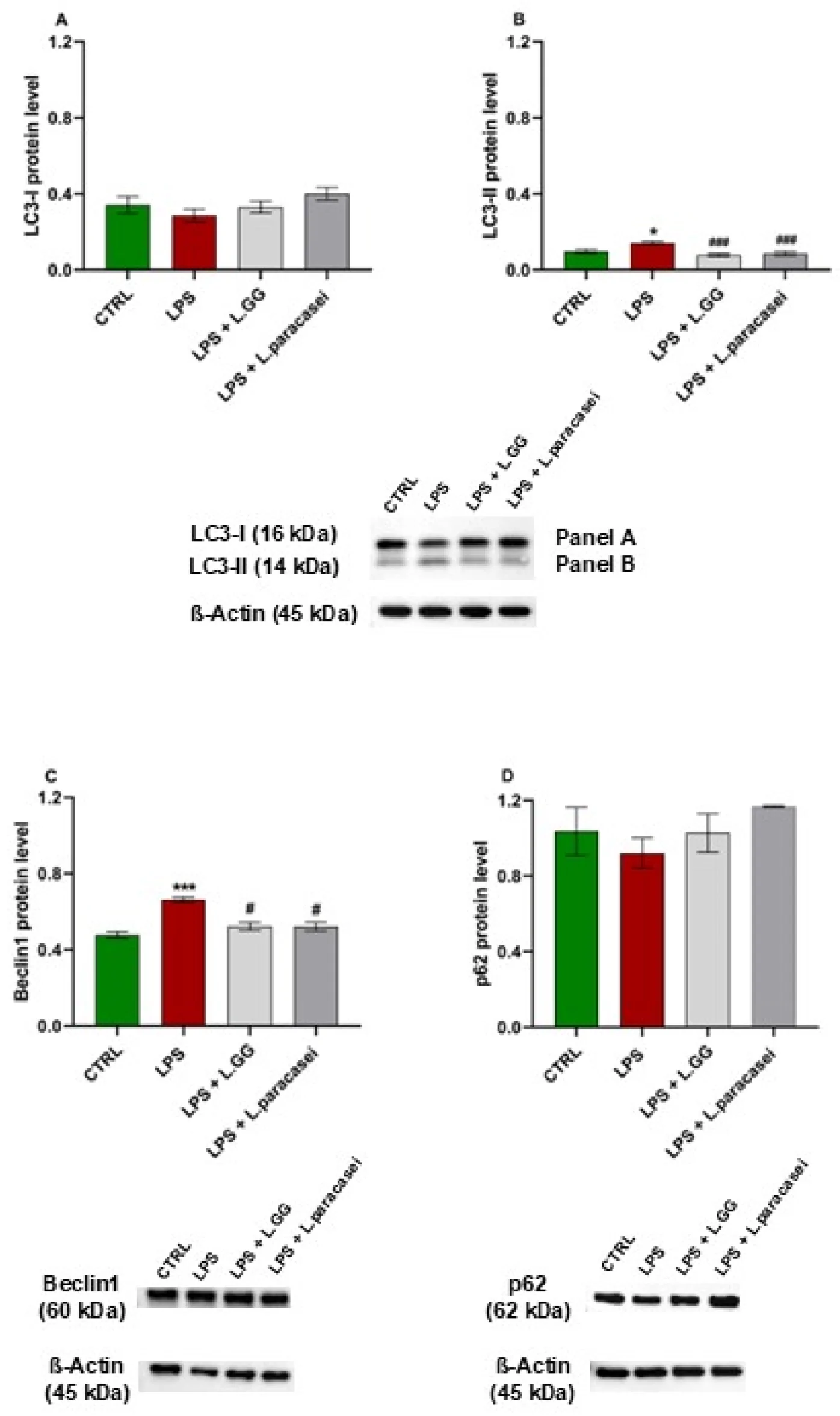
Western blot analysis of LC3-I (**A**), LC3-II (**B**), Beclin1 (**C**) and p62 (**D**) proteins in Caco-2 cells, untreated (CTRL), treated with LPS 10 μg/mL (LPS), LPS 10 μg/mL + LGG 10^8^ CFU/mL (LPS + LGG), LPS 10 μg/mL + *L. paracasei* 10^8^ CFU/mL (LPS + *L. paracasei)*, for 24 h. All data represent the result of at least three experiments (mean ± SEM). * *p* < 0.05, *** *p* < 0.001 compared to control group; ^#^ *p* < 0.05, ^###^ *p* < 0.001 compared to LPS group.

To evaluate the effects of LPS exposure on autophagy, the levels of key autophagy markers: LC3-I, LC3-II, Beclin1, and p62, were assessed by Western blot analysis. As shown in Figure 7A, the protein level of LC3-I remained unchanged across all treatment groups. In contrast, LC3-II levels significantly increased by 45.8% in Caco-2 cells exposed to LPS for 24 h compared to untreated controls (Figure 7B). Co-administration of LPS with LGG or *L. paracasei* significantly reduced LC3-II levels by 45.2% and 39.9%, respectively, compared to the LPS-only group.

Beclin1 protein levels significantly increased by 38.4% in cells treated with LPS alone for 24 h compared to controls (Figure 7C). This effect resulted in a significant reduction in the presence of probiotics, by −20.9% and −21.1% for LGG and *L. paracasei*, respectively, compared to LPS-treated cells, indicating a counteractive effect of the probiotics on Beclin1 expression.

Finally, p62 protein levels remained unchanged across all experimental conditions (Figure 7D), suggesting an impairment of autophagic flux at the degradation stage, rather than increased autophagosome formation.

**Figure 8:**
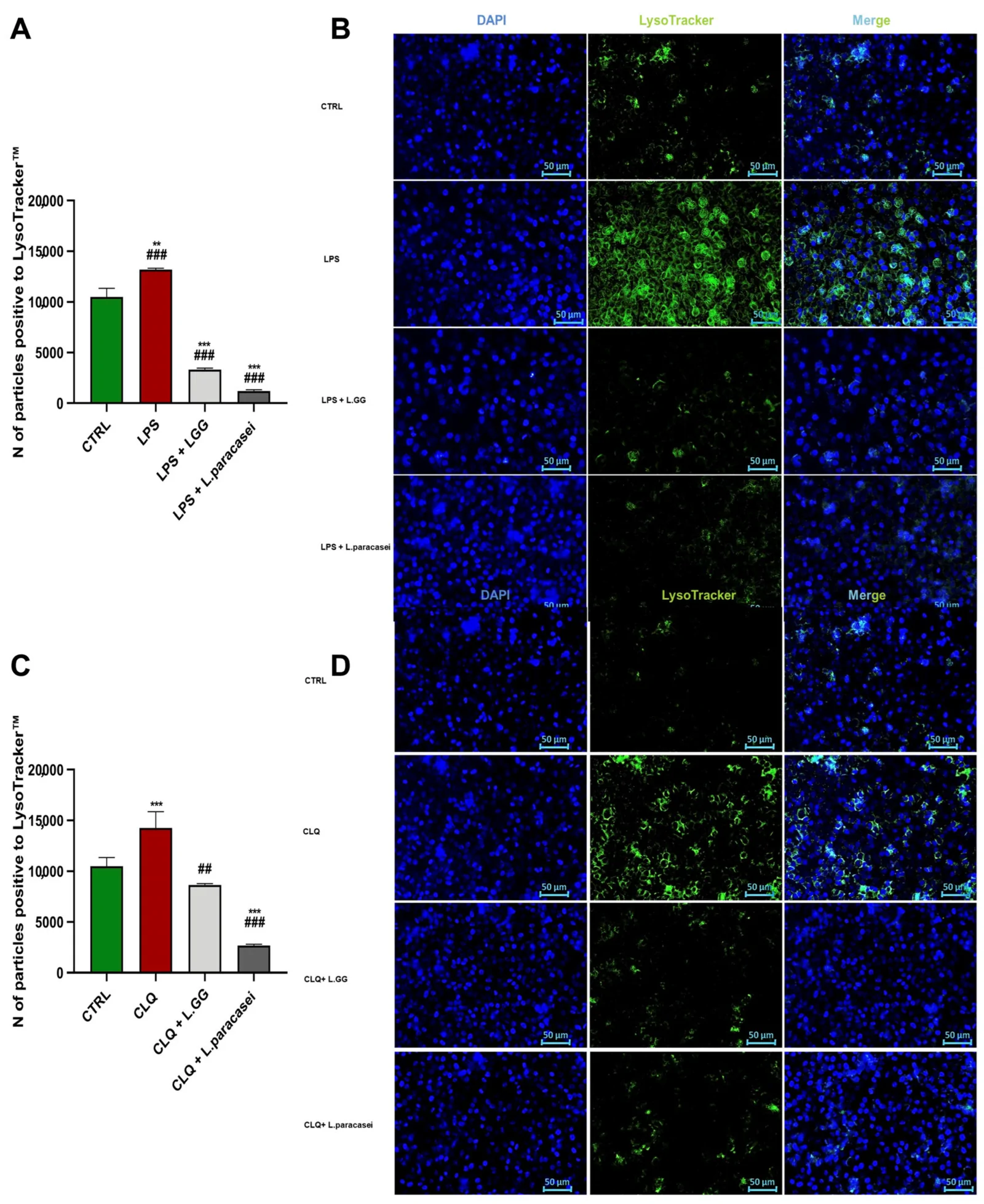
Quantitative and Qualitative Analysis of Lysosomal Compartments in Caco-2 cells after 24 h of treatment. (**A**,**C**) Quantitative assessment of lysosomal staining on Caco-2 cells, untreated (CTRL), treated with LPS 10 μg/mL (LPS), LPS 10 μg/mL + LGG 10^8^ CFU/mL (LPS + LGG), LPS 10 μg/mL + *L. paracasei* 10^8^ CFU/mL (LPS + *L. paracasei*), for 24 h. Data are presented as the mean number of LysoTracker positive vesicles per cell or per defined area unit, as appropriate. Statistical comparisons were performed as specified in the corresponding captions. All data represent the result of at least three experiments (mean ± SEM). ** *p* < 0.01, *** *p* < 0.001 compared to control group; ^##^ *p* < 0.01, ^###^ *p* < 0.001 compared to LPS or CLQ group. (**B**,**D**) Representative fluorescence microscopy images of Caco-2 cells subjected to the following treatment conditions: Caco-2 cells untreated (CTRL), treated with CLQ 2.5 × 10^−5^ M (CLQ), CLQ 2.5 × 10^−5^ M + LGG 10^8^ CFU/mL (CLQ + LGG), CLQ 2.5 × 10^−5^ M + *L. paracasei* 10^8^ CFU/mL (CLQ + *L. paracasei*), for 24 h. Green fluorescence corresponds to LysoTracker-positive lysosomes, while blue fluorescence indicates DAPI-stained nuclei. All images were acquired with the same exposure settings to allow direct comparison between treatment groups. Scale bars: 50 μm.

In light of the above results, we examined the hypothesis of autophagic flux blockage following LPS treatment. The data are shown in Figure 8, which includes quantitative analyses (Panels A and C) and qualitative analyses (Panels B and D) of lysosomal compartments in Caco-2 cells after exposure to LPS, chloroquine (CLQ), or co-treatment with probiotic strains. Quantitative results indicated a significant increase in both the number and fluorescence intensity of LysoTracker™ Green-positive vesicles in cells treated with LPS or CLQ compared to untreated controls (* *p* < 0.05, *** *p* < 0.001) (Figure 8A,C). This buildup of lysosomal staining suggested impaired autophagic flux, consistent with a disruption of lysosomal degradation. The similar effect seen in LPS-treated cells suggested that LPS, like the lysosomal inhibitor CLQ, blocked autophagic progression by hindering the clearance of autolysosomal content. Co-treatment of LPS or CLQ with LGG or *L. paracasei* significantly decreased both the number and fluorescence intensity of LysoTracker-positive vesicles (^###^ *p* < 0.001 vs. LPS group) (Figure 8A,C). This reversal of lysosomal accumulation indicated that probiotic treatment helped restore autophagic flux, reducing LPS- and CLQ-induced lysosomal dysfunction. These findings highlight the capacity of probiotics to improve lysosomal clearance and support autophagic homeostasis during inflammatory or pharmacological stress.

Fluorescence microscopy images (Figure 8B,D) confirmed the quantitative findings. Cells treated with LPS or CLQ displayed intense, clustered green fluorescence, indicating lysosomal buildup. In contrast, cells co-treated with LPS or CLQ and probiotics exhibited a more diffuse and evenly spread lysosomal staining pattern, similar to that of untreated controls. Overall, these data support the conclusion that probiotic treatment effectively reduces the impairment of lysosomal function caused by LPS and CLQ.

### 2.7. Inflammatory Response

**Figure 9:**
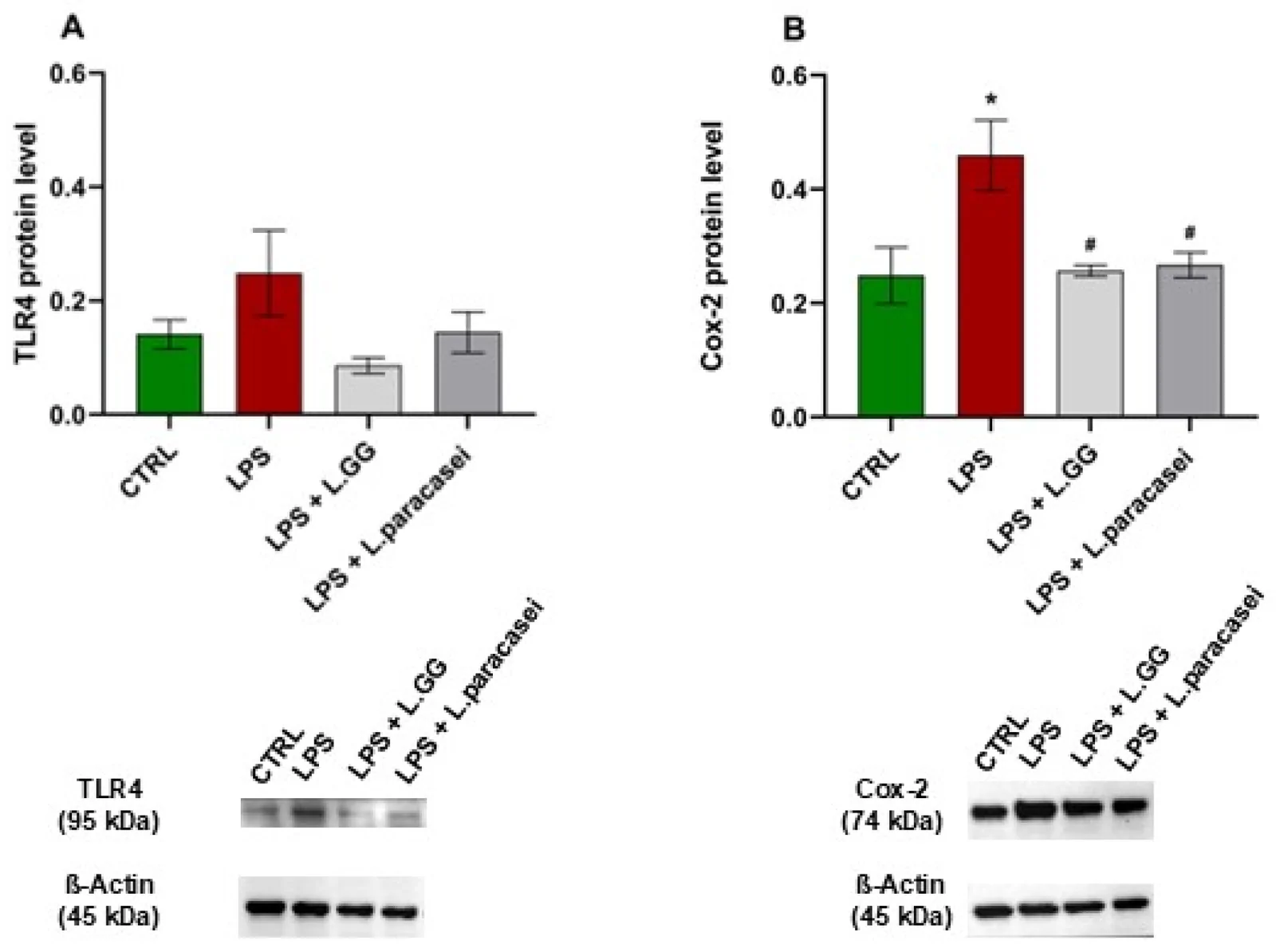
Western blot analysis of TLR4 (**A**) and Cox-2 (**B**) proteins in Caco-2 cells, untreated (CTRL), treated with LPS 10 μg/mL (LPS), LPS 10 μg/mL + LGG 10^8^ CFU/mL (LPS + LGG), LPS 10 μg/mL + *L. paracasei* 10^8^ CFU/mL (LPS + *L. paracasei*), for 24 h. All data represent the result of at least three experiments (mean ± SEM). * *p* < 0.05, compared to control group; ^#^ *p* < 0.05, compared to LPS group.

To examine the inflammatory response triggered by LPS treatment, the protein levels of TLR4 and cyclooxygenase-2 (Cox-2) were measured using Western blot analysis. As shown in Figure 9A, LPS administration increased TLR4 protein levels compared to untreated controls, although this increase was not statistically significant. Notably, co-treatment with LPS and either LGG or *L. paracasei* reversed this elevation, indicating a potential modulatory effect of these probiotic strains on TLR4 expression.

Additionally, LPS exposure significantly increased Cox-2 protein levels by 84.8% compared to untreated controls (Figure 9B). However, co-administration of LPS with LGG or *L. paracasei* significantly decreased the LPS-induced Cox-2 overexpression by 44.1% and 41.9%, respectively, compared to the LPS-only group. These results indicate that both probiotic strains may have anti-inflammatory effects by reducing the LPS-triggered rise in Cox-2 protein levels.

### 2.8. Apoptosis

**Figure 10:**
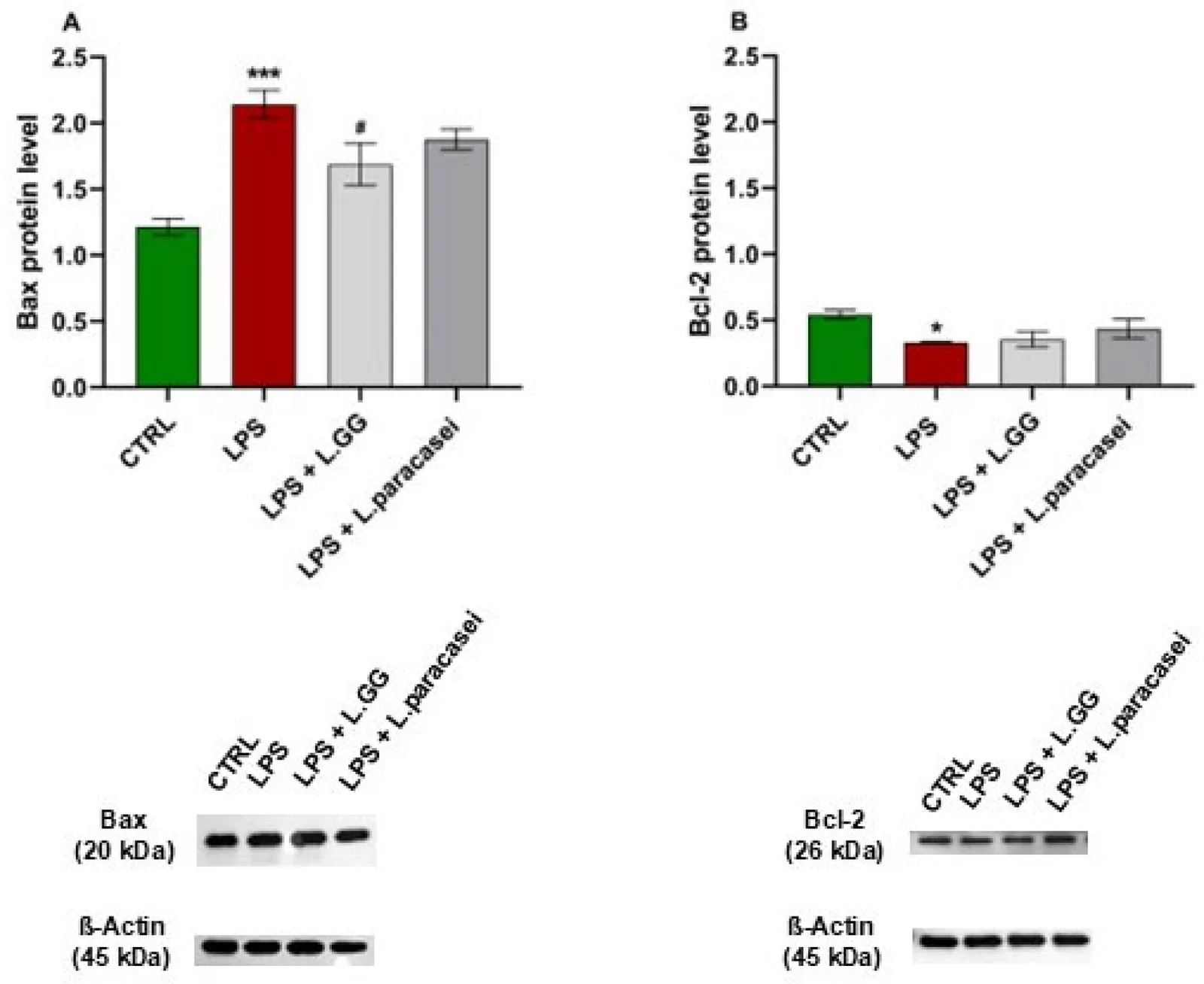
Western blot analysis of Bax (**A**) and Bcl-2 (**B**) proteins in Caco-2 cells, untreated (CTRL), treated with LPS 10 μg/mL (LPS), LPS 10 μg/mL + LGG 10^8^ CFU/mL (LPS + LGG), LPS 10 μg/mL + *L. paracasei* 10^8^ CFU/mL (LPS + *L. paracasei*), for 24 h. All data represent the result of at least three experiments (mean ± SEM). * *p* < 0.05, *** *p* < 0.001 compared to control group; ^#^ *p* < 0.05, compared to LPS group.

To examine whether LPS treatment triggers apoptosis, the levels of the pro-apoptotic protein Bax and the anti-apoptotic protein Bcl-2 were measured in Caco-2 cell extracts. As shown in Figure 10A, 24-h exposure to LPS significantly increased Bax levels by 76.7% compared to untreated cells. Co-treatment with LPS and LGG significantly decreased Bax levels by 21.3% compared to cells treated with LPS alone. In contrast, co-treatment with *L. paracasei* did not lead to a significant reduction in Bax levels.

Conversely, LPS exposure significantly reduced Bcl-2 levels by 50.1% compared to untreated controls (Figure 10B). Neither LGG nor *L. paracasei* co-administration significantly changed Bcl-2 levels relative to LPS-treated cells.

## 3. Discussion

In this study, we demonstrate that co-treatment with LGG or *L. paracasei* counteracts LPS-induced epithelial barrier dysfunction in Caco-2 cells by restoring tight junction protein expression, resolving autophagic flux blockade, and reducing inflammation and apoptosis.

The intestinal epithelium acts as a critical barrier, safeguarding the host from harmful antigens and pathogens [37]. Central to this protective function are tight junctions (TJs), which tightly regulate the passage of molecules between cells. When these junctions are disrupted by pathogenic bacteria or pro-inflammatory signals, intestinal permeability increases, fueling ongoing inflammation and contributing to disease development [38]. Among the numerous factors that compromise barrier integrity [39], lipopolysaccharide (LPS) an endotoxin produced by Gram-negative gut bacteria is particularly potent in driving epithelial dysfunction and inflammation [40,41]. LPS engages Toll-like receptors (TLRs) on innate immune cells, triggering signaling pathways that stimulate the release of pro-inflammatory cytokines and mediators, thereby exacerbating barrier breakdown and sustaining inflammatory processes.

Probiotics, especially Lactobacillus species, have shown promise in restoring intestinal barrier function and reducing inflammation, although their mechanisms of action are not fully understood. Lactobacillus species can positively influence the expression of TJ proteins and modulate inflammatory pathways during pathogen infection, supporting the assembly and redistribution of TJ proteins on the cell surface [5]. Additionally, these bacteria impact immune responses through TLR2-mediated pathways, either enhancing or suppressing the release of pro-inflammatory cytokines [42,43,44]. While they cannot directly activate TLR4, they can indirectly regulate its expression by reshaping the intestinal microbiota [45]. Exposure to inactivated Lactobacillus species can also activate macrophage inflammatory responses by inducing the production of pro-inflammatory mediators, including cytokines and reactive oxygen species (ROS), and by engaging key signaling pathways such as NF-κB and TLR2 [46]. For example, *L. plantarum* L15 has been shown to reduce dextran sulfate sodium (DSS)-induced inflammation by decreasing the expression of TLR4 and MyD88, as well as genes involved in the NF-κB pathway [47].

The present study investigated the role of two Lactobacillus strains (LGG and *L. paracasei*) in reducing LPS-induced epithelial barrier dysfunction using the Caco-2 cell line. Using TEER and FD4 assays, we found that LPS significantly compromised barrier integrity, shown by lower TEER values and increased FD4 permeability. While co-treatment with LGG or *L. paracasei* significantly restored the expression of key tight junction proteins (ZO-1 and Occludin), the functional assays (TEER and FD4 permeability) showed only non-significant trends toward improvement, possibly due to assay sensitivity or the co-treatment design [48,49].

The non-significant trends in TEER and FD4 might indicate a modest protective effect of the probiotics under these conditions, which may not be strong enough to reach statistical significance. This could also be due to assay variability: fluctuations in TEER and FD4 measurements (such as temperature, electrode placement, insert quality, and medium composition) might mask small effects. Additionally, simultaneous co-treatment with LPS and probiotics could limit their protective potential, while pre-treatment might more effectively improve barrier integrity.

The integrity of the intestinal barrier closely relates to the expression and placement of TJ proteins. In our study, the levels of key TJ proteins such as ZO-1 and Occludin were significantly decreased after LPS exposure but returned to normal following probiotic treatment. This result aligns with previous research, which shows that probiotics enhance TJ protein expression and facilitate their migration to the epithelial surface [5,50,51,52]. For example, *L. acidophilus* ATCC4356 increased ZO-1 and Occludin levels in both Caco-2 and HT-29 cells, helping to counteract damage caused by enteroinvasive *E. coli* 029:NM [50,51]. Likewise, *L. rhamnosus* MTCC-5897 competed with enterotoxigenic *E. coli* for binding sites on the epithelium and reversed damage to ZO-1, Claudin-1, Occludin, and Cingulin [52]. These results further highlight the potential of probiotics to support the epithelial barrier by regulating TJ proteins.

Previous studies have shown that LPS can trigger autophagy [12]. Autophagy is a highly conserved process where cytoplasmic components are enclosed in autophagosomes and transported to lysosomes for degradation and recycling. It serves as a vital survival mechanism that helps maintain cellular balance and the integrity of the cellular barrier. Under stress conditions, autophagy can be disrupted, especially when lysosomal function is impaired, leading to a blockage of autophagic flux. This disruption results in cellular dysfunction, energy depletion, and an inability to eliminate toxic substances [53,54,55].

Several pathways tightly regulate autophagy, especially the AKT/mTOR pathway, which influences cell survival, metabolism, and growth. Activation of receptor tyrosine kinases leads to the activation of PI3K/AKT and mTOR phosphorylation, which suppresses autophagy. Conversely, blocking the AKT/mTOR pathway is a key method of autophagy activation [19]. The expression of autophagy-related genes (e.g., Atg7, Atg9), the conversion of LC3-I to LC3-II, and the regulation of the Bcl-2/Beclin1 complex are also controlled by MAPK signaling pathways [56,57,58,59,60].

In our study, LPS-treated Caco-2 cells showed a significant decrease in phosphorylated AKT (p-AKT), along with a corresponding reduction in p-mTOR levels. They also displayed lower levels of phosphorylated ERK1/2, collectively indicating that LPS triggered autophagy activation. This was supported by increased levels of LC3-II and Beclin1, two key autophagy markers. However, the unchanged p62 levels suggest a blockade of autophagic flux, indicating that the accumulation of LC3-II and Beclin1 was due to impaired degradation of autophagic cargo at the autolysosome stage rather than increased production.

To evaluate autophagic flux, we measured the area of Lysotracker Green-positive acidic vesicles in cells treated with LPS and those co-treated with LPS and probiotics. These results were also compared with cells treated with chloroquine, a known inhibitor of autophagosome–lysosome fusion [60]. Immunofluorescence confirmed that LPS caused a blockage in autophagy flux, likely due to p-AKT inhibition. Importantly, this blockage represents a pathological impairment rather than a functional adaptive response. It is worth noting that not all probiotic strains modulate autophagy in the same way. For example, *L. reuteri* has been shown to inhibit autophagy in specific inflammatory contexts, potentially to prevent excessive self-digestion [61]. This strain-specific effect highlights that the restoration of autophagy flux, as observed with LGG and *L. paracasei*, is not a universal property of probiotics, but rather a targeted mechanism dependent on the bacterial strain and the nature of the stressor.

Probiotic co-treatment restored autophagy homeostasis, as evidenced by the normalization of LC3-II and Beclin1 levels. Immunofluorescence indicated that probiotics alleviated the lysosomal accumulation caused by LPS, consistent with restored autophagic flux. The partial restoration of p-AKT and p-ERK1/2 levels by probiotics may contribute to the reactivation of mTOR-dependent lysosomal biogenesis and acidification, thereby resolving the LPS-induced block in autophagosome–lysosome fusion. This hypothesis aligns with evidence that AKT/mTOR signaling regulates lysosomal v-ATPase assembly and cathepsin activity [62]. This recovery of flux, rather than just its induction, suggests a more advanced cytoprotective mechanism, where probiotics resolve a stalled process instead of simply activating it.

Considering the known crosstalk between inflammation and autophagy [62], we also examined TLR4 and Cox-2 levels. Their increase in LPS-treated cells indicated an inflammatory state that contributed to the disruption of autophagy. Conversely, probiotic co-treatment reduced Cox-2 levels, suggesting an anti-inflammatory effect that may have contributed to the restoration of autophagy. Overall, these results suggest that LPS induces cytotoxicity through autophagy dysregulation in the context of unresolved inflammation, while LGG and *L. paracasei* aid in reestablishing autophagy balance and cellular homeostasis, likely through anti-inflammatory mechanisms.

Yamoto et al. [63] emphasized the significance of controlling autophagosome buildup in intestinal injury, presenting it as a potential therapeutic target. Our data support the possible use of Lactobacilli in this area.

Additionally, LPS exposure caused apoptosis in Caco-2 cells, evidenced by increased Bax and decreased Bcl-2 protein levels. LGG co-treatment significantly countered these effects by lowering Bax expression, indicating that this strain may also influence apoptotic pathways, possibly through mitochondrial signaling or caspase inhibition mechanisms. Notably, co-administration with *L. paracasei* produced a similar reduction, although it was not statistically significant. The stronger anti-apoptotic effect of LGG compared to *L. paracasei* may reflect strain-specific differences in secreted metabolites (e.g., short-chain fatty acids, exopolysaccharides) or surface molecules (e.g., pili, lipoteichoic acid) that differentially engage host receptors such as TLR2 or GPR109A, as previously reported for LGG [29,64,65,66].

In summary, this study aimed to elucidate the protective mechanisms of LGG and *L. paracasei* against LPS-induced disruption of the epithelial barrier using an in vitro model.

Our findings demonstrate that LPS downregulates TJ protein expression, promotes inflammation, induces apoptosis and disrupts autophagic flux. Importantly, co-treatment with these probiotic strains effectively mitigates these effects, highlighting the central role of inflammation-driven autophagy impairment in LPS-induced intestinal dysfunction and supporting probiotic intervention as a promising strategy to restore mucosal homeostasis.

Despite these promising results, this study has some limitations. First, although the Caco-2 cell line is a well-established in vitro model for studying intestinal barrier function, it lacks the complexity of in vivo systems, including immune interactions and microbiota dynamics. Future research should validate these findings using animal models or organoid-based systems to confirm their translational relevance. Second, our research identifies autophagy flux restoration as a key pathway for barrier protection, highlighting the therapeutic potential of LGG and *L. paracasei*. However, it does not investigate which specific probiotic-derived compounds, metabolites, or receptor interactions are involved in modulating autophagy. This remains an important question for future mechanistic studies. Furthermore, we focused on short-term (24-h) responses; longer-term experiments are necessary to evaluate the persistence and clinical relevance of probiotic-mediated protection. Third, the lack of statistical significance in functional barrier assays (TEER and FD4), despite clear molecular evidence of TJ restoration, represents a key limitation. This discrepancy may stem from the inherent variability of functional assays, the timing of probiotic administration (co-treatment vs. pre-treatment), or the insensitivity of these methods to detect subtle but biologically relevant changes. Lastly, the clinical applicability of these findings should be explored in patient populations with conditions linked to barrier dysfunction, such as IBS or IBD.

## 4. Materials and Methods

### 4.1. Cell Culture and Treatments

Caco-2 cells (ATCC-HTB-37, derived from human colon adenocarcinoma) were cultured in Dulbecco’s Modified Eagle Medium (DMEM) supplemented with 10% (*v*/*v*) heat-inactivated fetal bovine serum (FBS), 1% (*v*/*v*) penicillin-streptomycin (10,000 U/mL penicillin and 10 mg/mL streptomycin), and 1% (*v*/*v*) non-essential amino acids (NEAA). Cells were maintained at 37 °C in a humidified atmosphere of 5% CO_2_. The culture medium was refreshed every 2–3 days, and cells were passaged at 80–90% confluence using 0.25% trypsin-EDTA. For the experiments, cells were seeded on transwell inserts (0.4 µm pore size) and allowed to differentiate for 21 days after reaching confluence, forming a polarized monolayer with functional tight junctions. For all experiments, cells were used at passages 5 and 15 to ensure the reproducibility and consistency of results. All experiments were performed in triplicate (technical replicates) and repeated in at least three independent biological replicates (n ≥ 3).

LPS from *Escherichia coli* O111:B4 (Sigma-Aldrich, Burlington, MA, USA) was used to induce epithelial barrier dysfunction. To evaluate the protective effects of probiotics, cells were co-incubated with *Lactobacillus rhamnosus GG* (LGG; ATCC 53103) or *Lactobacillus paracasei* (*L. paracasei* strain IMPC2.1) provided by the Institute of Sciences of Food Production, National Research Council (CNR), Bari, Italy, at concentrations ranging from 10^2^ to 10^8^ colony-forming units per milliliter (CFU/mL). The probiotic doses were selected based on dose-response studies that confirmed their efficacy in maintaining epithelial integrity, as well as our preliminary data. Each experimental condition included a corresponding control group of untreated cells cultured under identical conditions. The concentration of 10^8^ CFU/mL was confirmed to maintain stable bacterial viability throughout the 24-h co-incubation period, as shown in Supplementary Figure S1 (*p* > 0.05 vs. baseline), in line with recent findings on probiotic stability in epithelial co-culture models [24].

While recent taxonomic revisions have reclassified some Lactobacillus species into new genera (e.g., *Lacticaseibacillus*), the two strains mentioned above are cited according to their original and widely accepted designations in probiotic research.

### 4.2. Culture of Lactobacillus rhamnosus GG and Lactobacillus paracasei Strains

LGG and *L. paracasei* strains were cultured on de Man, Rogosa, and Sharpe (MRS) agar (Sigma, St. Louis, MO, USA, catalog number 69966) at 37 °C for 24 h under anaerobic conditions (5% CO_2_ in a sealed anaerobic chamber or using anaerobic gas packs). Bacterial cultures were harvested by centrifugation at 6000× *g* for 10 min at 4 °C, and the resulting pellet was washed three times with sterile phosphate-buffered saline (PBS, pH 7.4) to remove residual media components. The pellet was then re-suspended in sterile PBS, and the turbidity of the bacterial suspension was measured using the VITEK DensiCHEK (bioMérieux S.A., Marcy l’Étoile, France), according to the manufacturer’s instructions.

The instrument was calibrated against a McFarland turbidity standard prior to each measurement to ensure accuracy and reproducibility. The bacterial suspension was placed in a standard glass cuvette and inserted into the DensiCHEK device, which automatically provided a McFarland value. Suspensions were adjusted to the typically turbidity of 0.5 McFarland, corresponding to approximately 1 × 10^8^ CFU/mL. Then, The bacterial suspensions were adjusted to the desired CFU/mL concentrations through serial dilutions in unsupplemented DMEM (without FBS or antibiotics), which was then used for subsequent in vitro experiments. The turbidity of the bacterial suspension from the supernatant recovered after 24 h of treatment was measured to assess bacterial viability after treatment. The data, presented in the supplemental material (Figure S1), show that probiotic viability remained stable or slightly increased, with no statistically significant changes (*p* > 0.05). The experimental protocol included the removal of antibiotics during probiotic treatment, ensuring bacterial survival and a reliable assessment of their biological activity.

### 4.3. Caco-2 Cell Viability

The metabolic viability of Caco-2 cells in response to LPS and probiotic treatments was evaluated using the MTT assay (3-(4,5-dimethylthiazol-2-yl)-2,5-diphenyl-tetrazolium bromide). Caco-2 cells were seeded at a density of 4 × 10^4^ cells per well in 24-well culture plates and incubated overnight at 37 °C in a humidified 5% CO_2_ incubator to promote cell attachment. After 24 h, cells were treated with increasing concentrations of LPS (10–200 µg/mL), either alone or co-incubated with LGG or *L. paracasei* at a concentration of 10^8^ CFU/mL for 24 h. Following treatment, 50 µL of MTT solution (5 mg/mL in PBS) was added to each well, and the plates were incubated for 3 h at 37 °C in the dark to allow formazan crystal formation. The culture medium was then carefully aspirated, and 150 µL of dimethyl sulfoxide (DMSO) was added to each well to dissolve the formazan crystals. The absorbance of the dissolved formazan was measured at 570 nm using a microplate reader (FLUOstar Omega, BMG LABTECH, Ortenberg, Germany). The amount of formazan formed is directly proportional to the number of viable, metabolically active cells, and cellular viability was expressed as a percentage relative to untreated control cells.

### 4.4. Transepithelial Electrical Resistance Measurements

Trans-epithelial electrical resistance (TEER) measurements were conducted to evaluate trans-epithelial permeability. Caco-2 cells were cultured to 80–90% confluence in 75 cm^2^ flasks using DMEM supplemented with 10% (*v*/*v*) FBS and 1% (*v*/*v*) penicillin-streptomycin (10,000 U/mL penicillin and 10 mg/mL streptomycin). Cells were detached using 0.25% trypsin-EDTA and seeded at a density of 1 × 10^5^ cells/cm^2^ in 6-well plates containing PET Transwell^®^ inserts (Corning, 0.4 μm pore size, 4.67 cm^2^ growth area). Cultures were maintained at 37 °C in a humidified 5% CO_2_ incubator for 14 days, with medium changes every 2–3 days (2 mL in the basolateral compartment and 1.5 mL in the apical compartment). TEER measurements were taken on days 4, 7, 11, and 14 using an epithelial voltohmmeter (EVOM3, World Precision Instruments, Friedberg, Germany) to monitor the formation of a confluent monolayer with stable TEER values.

Once a confluent monolayer with stable TEER values was established, Caco-2 cells were treated with escalating concentrations of LPS (10–200 µg/mL) alone or co-incubated with *LGG* or *L. paracasei* at 10^8^ CFU/mL for 24 h. TEER measurements were performed at the end of the treatment period under both control and experimental conditions using the epithelial voltohmmeter. TEER values (Ω × cm^2^) were calculated using the formula:

TEER = (R − R_0_) × A

R is the measured resistance (Ω), R_0_ is the resistance of an empty insert without cells, and A is the effective membrane surface area (4.67 cm^2^ for a 6-well Transwell^®^ plate). All experiments were performed in triplicate, and data are presented as the mean ± standard error of the mean (SEM).

### 4.5. Paracellular Permeability

To evaluate the impact of LPS and the potential protective effects of LGG and *L. paracasei* on paracellular permeability, the permeability of the Caco-2 monolayer was measured using fluorescein isothiocyanate-labeled 4 kDa dextran (FD4; Sigma-Aldrich, Zwijndrecht, The Netherlands), as described in earlier studies [67]. Briefly, FD4 was dissolved in phenol red-free culture medium and applied to the apical side of the transwell system at a final concentration of 1 mg/mL. Basolateral samples (100 μL in triplicate) were collected at baseline (before FD4 application) and 2 h later to assess the translocation of FD4 across the monolayer.

The fluorescence intensity of the basolateral samples was measured using a Tecan Infinite 200 microplate reader, with excitation and emission wavelengths set at 485 nm and 535 nm, respectively. To find the FD4 concentration in the samples, fluorescence readings were compared to a standard curve created by serially diluting FD4 in a phenol red-free culture medium. The standard curve included these concentrations: 10 µg/mL, 7.5 µg/mL, 5 µg/mL, 2.5 µg/mL, 1 µg/mL, 0.75 µg/mL, 0.5 µg/mL, 0.25 µg/mL, 0.1 µg/mL, 0.05 µg/mL, and 0.01 µg/mL.

The concentration of FD4 in the basolateral compartment was expressed in ng/mL and normalized to baseline levels. The percentage change in FD4 concentration was calculated relative to the untreated control group (CTRL). Data are shown as the mean percentage change ± SEM from at least three independent experiments conducted in triplicate.

### 4.6. Western Blotting Analysis

To evaluate the effects of LPS and the potential protective roles of LGG and *L. paracasei* on TJ protein expression, inflammatory responses, autophagy, apoptosis markers, and their associated signaling pathways, Western blot analyses were performed. Untreated control and treated cells were collected, and proteins were extracted using Pierce RIPA lysis buffer (Thermo Scientific, Waltham, MA, USA) with added protease and phosphatase inhibitors. After homogenization, lysates were centrifuged at 14,000× *g* for 20 min at 4 °C, and the supernatant was used to measure protein levels. Protein concentrations were determined using the Bradford assay (Bio-Rad, Hercules, CA, USA), with bovine serum albumin (BSA) serving as the standard. Aliquots containing 80 µg of protein were denatured with 4× Laemmli sample buffer at 70 °C for 10 min and separated by SDS-PAGE on 4–12% gradient polyacrylamide gels (Bio-Rad). Proteins were transferred onto PVDF membranes via a semi-dry transfer system. Membranes were blocked with 5% non-fat dry milk in Tris-buffered saline with 0.1% Tween-20 (TBST) for 1 h at room temperature, then incubated overnight at 4 °C with primary antibodies against ZO-1, Occludin, Claudin-1, AKT, p-AKT, mTOR, p-mTOR, ERK1/2, p-ERK1/2, LC3 I/II, Beclin1, p62, TLR4, Cox-2, Bax, and Bcl-2. After washing with TBST, membranes were incubated with HRP-conjugated secondary antibodies (anti-rabbit IgG or anti-mouse IgG) for 1 h at room temperature. Protein bands were visualized using Clarity Western ECL substrate (Bio-Rad) and imaged with a ChemiDoc Imaging System (Bio-Rad). Band intensities were quantified with Image Lab software 6.1 (Bio-Rad) and normalized to β-actin for densitometry.

### 4.7. Assessment of Autophagic Flux via Lysosomal Labeling

To measure autophagic flux, lysosomal compartments were stained with LysoTracker™ Green DND-26 (L7526, Invitrogen, Molecular Probes, Eugene, OR, USA), a fluorescent dye that specifically accumulates in acidic organelles like lysosomes [68]. After treatments, including stimulation with LPS alone or with selected probiotic strains, LysoTracker Green was directly added to the culture medium at a final concentration of 5 nM. Cells were incubated with the dye for 20 min at room temperature in the dark to ensure proper uptake and to minimize photobleaching. After staining, cells were gently washed three times with phosphate-buffered saline (PBS) to remove excess dye and reduce background fluorescence.

To enable nuclear visualization and facilitate the identification of individual cells, a counterstain was performed using 4′,6-diamidino-2-phenylindole (DAPI; Thermo Fisher Scientific, Waltham, MA, USA), applied in accordance with the manufacturer’s protocol. After staining procedures were completed, coverslips were mounted onto glass slides using an anti-fade mounting medium (P10144, ProLong™ Gold Antifade Mountant, Thermo Fisher Scientific, Waltham, MA, USA) to preserve fluorescence integrity and prevent signal degradation during image acquisition.

To evaluate the inhibition of autophagic flux, chloroquine (CLQ) (J64459.22, Thermo Fisher Scientific, Waltham, MA, USA), a well-characterized lysosomal inhibitor, was used as a positive control. CLQ was applied at a final concentration of 2.5 × 10^−5^ M, either alone or in combination with probiotic treatments, to confirm the accumulation of autophagosomes due to impaired lysosomal degradation.

Fluorescence imaging was conducted using a Nikon Eclipse Ti2 inverted fluorescence microscope (Nikon Instruments Inc., Tokyo, Japan), equipped with a high-resolution CCD digital camera (DP70; Olympus, Tokyo, Japan) to ensure high-quality image capture. Fluorescent signals were excited using Krypton Argon (Kr-Ar) and Argon (Ar) lasers, with excitation/emission filters optimized for LysoTracker™ Green and DAPI. Specifically, LysoTracker Green was excited at 488 nm and detected through a green emission channel, while DAPI was excited at 358 nm and detected through a blue emission channel. All imaging was performed using a 20× objective lens to balance resolution and field coverage.

To ensure consistency and accurate quantification, all images were captured using standardized exposure settings for each fluorescence channel across all experimental groups. This method reduced variability caused by imaging parameters, resulting in a dependable comparison of fluorescence intensities.

For each experimental condition, fluorescence images were collected from five to ten randomly selected, non-overlapping fields of view per sample to obtain representative data. Acquired images were analyzed using ImageJ 1.54m (National Institutes of Health, Bethesda, MD, USA), an open-source, interactive image processing software. Quantitative analysis included measuring fluorescence intensity and counting LysoTracker-positive vesicular structures (lysosomes), which were performed using threshold-based particle analysis and region-of-interest (ROI) tools within the software. All quantitative data obtained from image analysis are presented as mean ± SEM, based on measurements from both biological and technical replicates.

### 4.8. Statistical Analysis

Statistical analysis was conducted using R Studio (version 4.1.2, R Foundation for Statistical Computing, Vienna, Austria). Statistical significance was set at *p* < 0.05. All data are shown as the mean ± SEM from at least three independent experiments performed in triplicate. Given the subdivision of the experimental groups and the different treatments, the data obtained are independent; therefore, the statistical tests applied are unpaired. For data that follow a normal distribution, differences between groups were tested with a one-way analysis of variance (ANOVA), followed by Tukey’s post hoc test for multiple comparisons. When data did not meet the normality assumption, as confirmed by the Shapiro-Wilk test, the non-parametric Kruskal-Wallis test was used, followed by Dunn’s post hoc test for pairwise comparisons.

## 5. Conclusions

In conclusion, our study demonstrates that LGG and *L. paracasei* decrease LPS-induced epithelial barrier dysfunction by reducing inflammation and restoring TJ protein expression. These findings highlight the potential of probiotics as therapeutic or supplementary agents for conditions involving intestinal barrier disruption, such as inflammatory bowel diseases.

## Data Availability

The Raw data are available in the link “https://doi.org/10.6084/m9.figshare.28505366”, 11 June 2025.

## Supplementary Materials

The following supporting information can be downloaded at: https://www.mdpi.com/article/10.3390/ijms262211148/s1.

## References

[1] Ghosh S., Whitley C.S., Haribabu B., Jala V.R. (2021). Regulation of Intestinal Barrier Function by Microbial Metabolites. Cell Mol. Gastroenterol. Hepatol..

[2] Horowitz A., Chanez-Paredes S.D., Haest X., Turner J.R. (2023). Paracellular Permeability and Tight Junction Regulation in Gut Health and Disease. Nat. Rev. Gastroenterol. Hepatol..

[3] Arumugam P., Saha K., Nighot P. (2025). Intestinal Epithelial Tight Junction Barrier Regulation by Novel Pathways. Inflamm. Bowel Dis..

[4] Sanders M.E., Merenstein D.J., Reid G., Gibson G.R., Rastall R.A. (2019). Probiotics and Prebiotics in Intestinal Health and Disease: From Biology to the Clinic. Nat. Rev. Gastroenterol. Hepatol..

[5] Bai Y., Lyu M., Fukunaga M., Watanabe S., Iwatani S., Miyanaga K., Yamamoto N. (2022). *Lactobacillus johnsonii* Enhances the Gut Barrier Integrity via the Interaction Between GAPDH and the Mouse Tight Junction Protein JAM-2. Food Funct..

[6] Elkholy S.E., Maher S.A., Abd El-Hamid N.R., Elsayed H.A., Hassan W.A., Abdelmaogood A.K.K., Hussein S.M., Jaremko M., Alshawwa S.Z., Alharbi H.M. (2023). The Immunomodulatory Effects of Probiotics and Azithromycin in Dextran Sodium Sulfate-Induced Ulcerative Colitis in Rats via TLR4-NF-kB and P38-MAPK Pathway. Biomed. Pharmacother..

[7] Kumar P., Schroder E.A., Rajaram M.V.S., Harris E.N., Ganesan L.P. (2024). The Battle of LPS Clearance in Host Defense vs. Inflammatory Signaling. Cells.

[8] Candelli M., Franza L., Pignataro G., Ojetti V., Covino M., Piccioni A., Gasbarrini A., Franceschi F. (2021). Interaction between Lipopolysaccharide and Gut Microbiota in Inflammatory Bowel Diseases. Int. J. Mol. Sci..

[9] Xiao X., Cheng Y., Fu J., Lu Z., Wang F., Jin M., Zong X., Wang Y. (2021). Gut Immunity and Microbiota Dysbiosis Are Associated with Altered Bile Acid Metabolism in LPS-Challenged Piglets. Oxid. Med. Cell Longev..

[10] Di Vincenzo F., Del Gaudio A., Petito V., Lopetuso L.R., Scaldaferri F. (2024). Gut microbiota, intestinal permeability, and systemic inflammation: A narrative review. Intern. Emerg. Med..

[11] Luo R., Yao Y., Chen Z., Sun X. (2025). An examination of the LPS-TLR4 immune response through the analysis of molecular structures and protein-protein interactions. Cell Commun. Signal..

[12] Han C., Ding Z., Shi H., Qian W., Hou X., Lin R. (2016). The Role of Probiotics in Lipopolysaccharide-Induced Autophagy in Intestinal Epithelial Cells. Cell Physiol. Biochem..

[13] Li W., He P., Huang Y., Li Y.F., Lu J., Li M., Kurihara H., Luo Z., Meng T., Onishi M. (2021). Selective Autophagy of Intracellular Organelles: Recent Research Advances. Theranostics.

[14] Deretic V. (2021). Autophagy in Inflammation, Infection, and Immunometabolism. Immunity.

[15] Lu G., Wang Y., Shi Y., Zhang Z., Huang C., He W., Wang C., Shen H.M. (2022). Autophagy in Health and Disease: From Molecular Mechanisms to Therapeutic Target. MedComm.

[16] Larabi A., Barnich N., Nguyen H.T.T. (2020). New Insights into the Interplay between Autophagy, Gut Microbiota and Inflammatory Responses in IBD. Autophagy.

[17] Miceli C., Leri M., Stefani M., Bucciantini M. (2023). Autophagy-Related Proteins: Potential Diagnostic and Prognostic Biomarkers of Aging-Related Diseases. Ageing Res. Rev..

[18] Liu W.J., Ye L., Huang W.F., Guo L., Xu Z.G., Wu H.L., Yang C., Liu H.F. (2016). p62 Links the Autophagy Pathway and the Ubiquitin-Proteasome System upon Ubiquitinated Protein Degradation. Cell Mol. Biol. Lett..

[19] Li R., Chai L., Lei L., Guo R., Wen X. (2023). CDKL3 Promotes Non-Small Cell Lung Cancer by Suppressing Autophagy via Activation of PI3K/Akt/mTOR Pathway. Mol. Biotechnol..

[20] La Rosa F., Zoia C.P., Bazzini C., Bolognini A., Saresella M., Conti E., Ferrarese C., Piancone F., Marventano I., Galimberti D. (2022). Modulation of MAPK- and PI3/AKT-Dependent Autophagy Signaling by Stavudine (D4T) in PBMC of Alzheimer’s Disease Patients. Cells.

[21] Liu L., Jin R., Hao J., Zeng J., Yin D., Yi Y., Zhu M., Mandal A., Hua Y., Ng C.K. (2020). Consumption of the Fish Oil High-Fat Diet Uncouples Obesity and Mammary Tumor Growth through Induction of Reactive Oxygen Species in Protumor Macrophages. Cancer Res..

[22] Jyoti, Dey P. (2025). Mechanisms and implications of the gut microbial modulation of intestinal metabolic processes. NPJ Metab. Health Dis..

[23] Park I., Mannaa M. (2025). Fermented Foods as Functional Systems: Microbial Communities and Metabolites Influencing Gut Health and Systemic Outcomes. Foods.

[24] Zhang Y., Li Y., Ren X., Zhang X., Wu Z., Liu L. (2023). The Positive Correlation of Antioxidant Activity and Prebiotic Effect about Oat Phenolic Compounds. Food Chem..

[25] Nemati M., Omrani G.R., Ebrahimi B., Montazeri-Najafabady N. (2021). The Beneficial Effects of Probiotics via Autophagy: A Systematic Review. Biomed. Res. Int..

[26] Cui Y., Liu L., Dou X., Wang C., Zhang W., Gao K., Liu J., Wang H. (2017). *Lactobacillus reuteri* ZJ617 Maintains Intestinal Integrity via Regulating Tight Junction, Autophagy and Apoptosis in Mice Challenged with Lipopolysaccharide. Oncotarget.

[27] Engevik M.A., Luk B., Chang-Graham A.L., Hall A., Herrmann B., Ruan W., Endres B.T., Shi Z., Garey K.W., Hyser J.M. (2019). Bifidobacterium dentium Fortifies the Intestinal Mucus Layer via Autophagy and Calcium Signaling Pathways. MBio.

[28] Lu R., Shang M., Zhang Y.G., Jiao Y., Xia Y., Garrett S., Bakke D., Bauerl C., Martinez G.P., Kim C.H. (2020). Lactic Acid Bacteria Isolated from Korean Kimchi Activate the Vitamin D Receptor-autophagy Signaling Pathways. Inflamm. Bowel Dis..

[29] Orlando A., Linsalata M., Notarnicola M., Tutino V., Russo F. (2014). *Lactobacillus* GG Restoration of The Gliadin Induced Epithelial Barrier Disruption: The Role of Cellular Polyamines. BMC Microbiol..

[30] Orlando A., Linsalata M., D’attoma B., Russo F. (2017). Changes in Paracellular Permeability Induced by Pepsin-Trypsin Digested Gliadin (PTG): Role of Polyamines in the *Lactobacillus rhamnosus* GG Protective Action. J. Funct. Foods.

[31] Riezzo G., Orlando A., D’Attoma B., Guerra V., Valerio F., Lavermicocca P., De Candia S., Russo F. (2012). Randomised Clinical Trial: Efficacy of *Lactobacillus paracasei*-Enriched Artichokes in the Treatment of Patients with Functional Constipation—A Double-Blind, Controlled, Crossover Study. Aliment. Pharmacol. Ther..

[32] Orlando A., Refolo M.G., Messa C., Amati L., Lavermicocca P., Guerra V., Russo F. (2012). Antiproliferative and Proapoptotic Effects of Viable or Heat-Killed *Lactobacillus paracasei* IMPC2.1 and *Lactobacillus rhamnosus* GG in HGC-27 Gastric and DLD-1 Colon Cell Lines. Nutr. Cancer.

[33] Panse N., Gerk P.M. (2022). The Caco-2 Model: Modifications and Enhancements to Improve Efficiency and Predictive Performance. Int. J. Pharm..

[34] Chen Y., Li Y., Li X., Fang Q., Li F., Chen S., Chen W. (2024). Indole-3-propionic acid alleviates intestinal epithelial cell injury via regulation of the TLR4/NF-κB pathway to improve intestinal barrier function. Mol. Med. Rep..

[35] Liu L., Chen T., Xie Z., Zhang Y., He C., Huang Y. (2024). Butyric acid alleviates LPS-induced intestinal mucosal barrier damage by inhibiting the RhoA/ROCK2/MLCK signaling pathway in Caco2 cells. PLoS ONE.

[36] He S., Guo Y., Zhao J., Xu X., Wang N., Liu Q. (2020). Ferulic Acid Ameliorates Lipopolysaccharide-Induced Barrier Dysfunction via MicroRNA-200c-3p-Mediated Activation of PI3K/AKT Pathway in Caco-2 Cells. Front. Pharmacol..

[37] Al-Sadi R., Nighot P., Nighot M., Haque M., Rawat M., Ma T.Y. (2021). *Lactobacillus acidophilus* Induces a Strain-specific and Toll-Like Receptor 2-Dependent Enhancement of Intestinal Epithelial Tight Junction Barrier and Protection Against Intestinal Inflammation. Am. J. Pathol..

[38] Bhat A.A., Uppada S., Achkar I.W., Hashem S., Yadav S.K., Shanmugakonar M., Al-Naemi H.A., Haris M., Uddin S. (2018). Tight Junction Proteins and Signaling Pathways in Cancer and Inflammation: A Functional Crosstalk. Front. Physiol..

[39] Celebi Sözener Z., Cevhertas L., Nadeau K., Akdis M., Akdis C.A. (2020). Environmental Factors in Epithelial Barrier Dysfunction. J. Allergy Clin. Immunol..

[40] Singh S., Bhatia R., Singh A., Singh P., Kaur R., Khare P., Purama R.K., Boparai R.K., Rishi P., Ambalam P. (2018). Probiotic Attributes and Prevention of LPS-Induced Pro-Inflammatory Stress in RAW264.7 Macrophages and Human Intestinal Epithelial Cell Line (Caco-2) by Newly Isolated Weissella Cibaria Strains. Food Funct..

[41] Ghosh S.S., Wang J., Yannie P.J., Ghosh S. (2020). Intestinal Barrier Dysfunction, LPS Translocation, and Disease Development. J. Endocr. Soc..

[42] Morishita M., Sagayama R., Yamawaki Y., Yamaguchi M., Katsumi H., Yamamoto A. (2022). Activation of Host Immune Cells by Probiotic-Derived Extracellular Vesicles via TLR2-Mediated Signaling Pathways. Biol. Pharm. Bull..

[43] Wang J., Zhang W., Wang S., Liu H., Zhang D., Wang Y., Ji H. (2019). Swine-Derived Probiotic *Lactobacillus plantarum* Modulates Porcine Intestinal Endogenous Host Defense Peptide Synthesis Through TLR2/MAPK/AP-1 Signaling Pathway. Front. Immunol..

[44] Lee J., Jung I., Choi J.W., Lee C.W., Cho S., Choi T.G., Sohn M., Park Y.I. (2019). Micronized and Heat-Treated *Lactobacillus plantarum* LM1004 Stimulates Host Immune Responses via the TLR-2/MAPK/NF-kappaB Signalling Pathway In Vitro and In Vivo. J. Microbiol. Biotechnol..

[45] Wang W., Li Y., Han G., Li A., Kong X. (2022). *Lactobacillus fermentum* CECT5716 Alleviates the Inflammatory Response in Asthma by Regulating TLR2/TLR4 Expression. Front. Nutr..

[46] Rocha-Ramirez L.M., Perez-Solano R.A., Castanon-Alonso S.L., Moreno Guerrero S.S., Ramirez Pacheco A., Garcia Garibay M., Eslava C. (2017). Probiotic *Lactobacillus* Strains Stimulate the Inflammatory Response and Activate Human Macrophages. J. Immunol. Res..

[47] Yu P., Ke C., Guo J., Zhang X., Li B. (2020). *Lactobacillus plantarum* L15 Alleviates Colitis by Inhibiting LPS-Mediated NF-kappaB Activation and Ameliorates DSS-Induced Gut Microbiota Dysbiosis. Front. Immunol..

[48] Li Y., Zhang Y., Dong L., Li Y., Liu Y., Liu Y., Liu L., Liu L. (2024). Fermentation of *Lactobacillus fermentum* NB02 with Feruloyl Esterase Production Increases the Phenolic Compounds Content and Antioxidant Properties of Oat Bran. Food Chem..

[49] Li Y., Shi R., Qin C., Zhang Y., Liu L. (2021). Gluten-free and prebiotic oat bread: Optimization formulation by transglutaminase improvement dough structure. J. Food Process. Preserv..

[50] Resta-Lenert S., Barrett K.E. (2003). Live Probiotics Protect Intestinal Epithelial Cells from The Effects of Infection with Enteroinvasive *Escherichia coli* (EIEC). Gut.

[51] Resta-Lenert S., Barrett K.E. (2006). Probiotics and Commensals Reverse TNF-Alpha- and IFN-Gamma-Induced Dysfunction in Human Intestinal Epithelial Cells. Gastroenterology.

[52] Bhat M.I., Sowmya K., Kapila S., Kapila R. (2020). Potential Probiotic *Lactobacillus rhamnosus* (MTCC-5897) Inhibits *Escherichia coli* Impaired Intestinal Barrier Function by Modulating the Host Tight Junction Gene Response. Probiotics Antimicrob. Proteins.

[53] Neal M.D., Sodhi C.P., Dyer M., Craig B.T., Good M., Jia H., Yazji I., Afrazi A., Richardson W.M., Beer-Stolz D. (2013). A Critical Role for TLR4 Induction of Autophagy in the Regulation of Enterocyte Migration and the Pathogenesis of Necrotizing Enterocolitis. J. Immunol..

[54] Cheng H.J., Hsu W.L., Lin P., Chen Y.C., Lin T.H., Fang S.S., Tsai M.H., Lin Y.J., Wang S.P., Chen H. (2024). Involvement of Autophagy and Gut Dysbiosis in Ambient Particulate Matter-Induced Colonic Inflammation. Ecotoxicol. Environ. Saf..

[55] Button R.W., Roberts S.L., Willis T.L., Hanemann C.O., Luo S. (2017). Accumulation of Autophagosomes Confers Cytotoxicity. J. Biol. Chem..

[56] Fan J., Ren D., Wang J., Liu X., Zhang H., Wu M., Yang G. (2020). Bruceine D Induces Lung Cancer Cell Apoptosis and Autophagy via the ROS/MAPK Signaling Pathway In Vitro and In Vivo. Cell Death Dis..

[57] Kim N.Y., Mohan C.D., Sethi G., Ahn K.S. (2024). Cannabidiol Activates MAPK Pathway to Induce Apoptosis, Paraptosis, and Autophagy in Colorectal Cancer Cells. J. Cell Biochem..

[58] Ren S., Chen Y., Wang Q., Song L., Xin Z., Shi M., Liu X. (2024). NUPR1 Induces Autophagy and Promotes the Rogression of Esophageal Squamous Cell Carcinoma via the MAPK-mTOR Pathway. Pathol. Res. Pract..

[59] Cao W., Li J., Yang K., Cao D. (2021). An Overview of Autophagy: Mechanism, Regulation and Research Progress. Bull. Cancer.

[60] Mauthe M., Orhon I., Rocchi C., Zhou X., Luhr M., Hijlkema K.J., Coppes R.P., Engedal N., Mari M., Reggiori F. (2018). Chloroquine Inhibits Autophagic Flux by Decreasing Autophagosome-Lysosome Fusion. Autophagy.

[61] Haririzadeh Jouriani F., Torfeh M., Torkamaneh M., Sepehr A., Rohani M., Aghamohammad S. (2024). The Preventive and Therapeutic Role of *Lactobacillus* spp. in In Vitro Model of Inflammation via Affecting Autophagy Signaling Pathway. Immun. Inflamm. Dis..

[62] Zhou M., Xu W., Wang J., Yan J., Shi Y., Zhang C., Ge W., Wu J., Du P., Chen Y. (2018). Boosting mTOR-dependent autophagy via upstream TLR4-MyD88-MAPK signalling and downstream NF-κB pathway quenches intestinal inflammation and oxidative stress injury. eBioMedicine.

[63] Yamoto M., Lee C., Chusilp S., Yazaki Y., Alganabi M., Li B., Pierro A. (2019). The Role of Autophagy in Intestinal Epithelial Injury. Pediatr. Surg. Int..

[64] Wang L., Xu Z., Bains A., Ali N., Shang Z., Patil A., Patil S. (2024). Exploring Anticancer Potential of *Lactobacillus* Strains: Insights into Cytotoxicity and Apoptotic Mechanisms on HCT 115 Cancer Cells. Biologics.

[65] Sharma S., Singh R.L., Kakkar P. (2011). Modulation of Bax/Bcl-2 and Caspases by Probiotics during Acetaminophen Induced Apoptosis in Primary Hepatocytes. Food Chem. Toxicol..

[66] Amin M., Navidifar T., Saeb S., Barzegari E., Jamalan M. (2023). Tumor-targeted Induction of Intrinsic Apoptosis in Colon Cancer Cells by *Lactobacillus plantarum* and *Lactobacillus rhamnosus* Strains. Mol. Biol. Rep..

[67] De Gregorio A., Serafino A., Krasnowska E.K., Superti F., Di Fazio M.R., Fuggetta M.P., Hammarberg Ferri I., Fiorentini C. (2023). Protective Effect of Limosilactobacillus fermentum ME-3 against the Increase in Paracellular Permeability Induced by Chemotherapy or Inflammatory Conditions in Caco-2 Cell Models. Int. J. Mol. Sci..

[68] Albrecht L.V., Tejeda-Muñoz N., De Robertis E.M. (2020). Protocol for Probing Regulated Lysosomal Activity and Function in Living Cells. STAR Protoc..

