# Correction: Orlando et al. *Lactobacillus rhamnosus* GG and *Lactobacillus paracasei* IMPC2.1 Mitigate LPS-Induced Epithelial Barrier Dysfunction: A Focus on Autophagy Regulation. *Int. J. Mol. Sci.* 2025, *26*, 11148

**DOI:** 10.3390/ijms27146493

**Published:** 2026-07-22

**Authors:** Antonella Orlando, Fatima Maqoud, Domenica Mallardi, Simona Drago, Eleonora Malerba, Guglielmina Chimienti, Francesco Russo

**Affiliations:** 1 Functional Gastrointestinal Disorders Research Group, National Institute of Gastroenterology IRCCS “S. de Bellis”, 70013 Castellana Grotte, Italy; antonella.orlando@irccsdebellis.it (A.O.); fatima.maqoud@irccsdebellis.it (F.M.); domenica.mallardi@irccsdebellis.it (D.M.); simona.drago@irccsdebellis.it (S.D.); eleonora.malerba@irccsdebellis.it (E.M.); 2 Department of Biosciences, Biotechnology and Environment, University di Bari “Aldo Moro”, 70100 Bari, Italy; guglielminaalessandra.chimienti@uniba.it

In the original publication [1], reference [41] has been retracted and therefore requires revision.

The previous reference:

41. Wang, Y.; Kong, D. MicroRNA-136 Promotes Lipopolysaccharide-Induced ATDC5 Cell Injury and Inflammatory Cytokine Expression by Targeting Myeloid Cell Leukemia 1. *J. Cell Biochem.* **2018**, *119*, 9316–9326.

has been replaced with:

41. Ghosh, S.S.; Wang, J.; Yannie, P.J.; Ghosh, S. Intestinal Barrier Dysfunction, LPS Translocation, and Disease Development. *J. Endocr. Soc.* **2020**, *4*, bvz039.

The authors state that the scientific conclusions are unaffected. This correction was approved by the Academic Editor. The original publication has also been updated.
