# Supplementary figures and images for: Lactobacillus rhamnosus GG and Lactobacillus paracasei IMPC2.1 Mitigate LPS-Induced Epithelial Barrier Dysfunction: A Focus on Autophagy Regulation
Source: Int J Mol Sci. 2025 Nov 18;26(22):11148. doi: 10.3390/ijms262211148 (PMC12652688; doi:10.3390/ijms262211148)

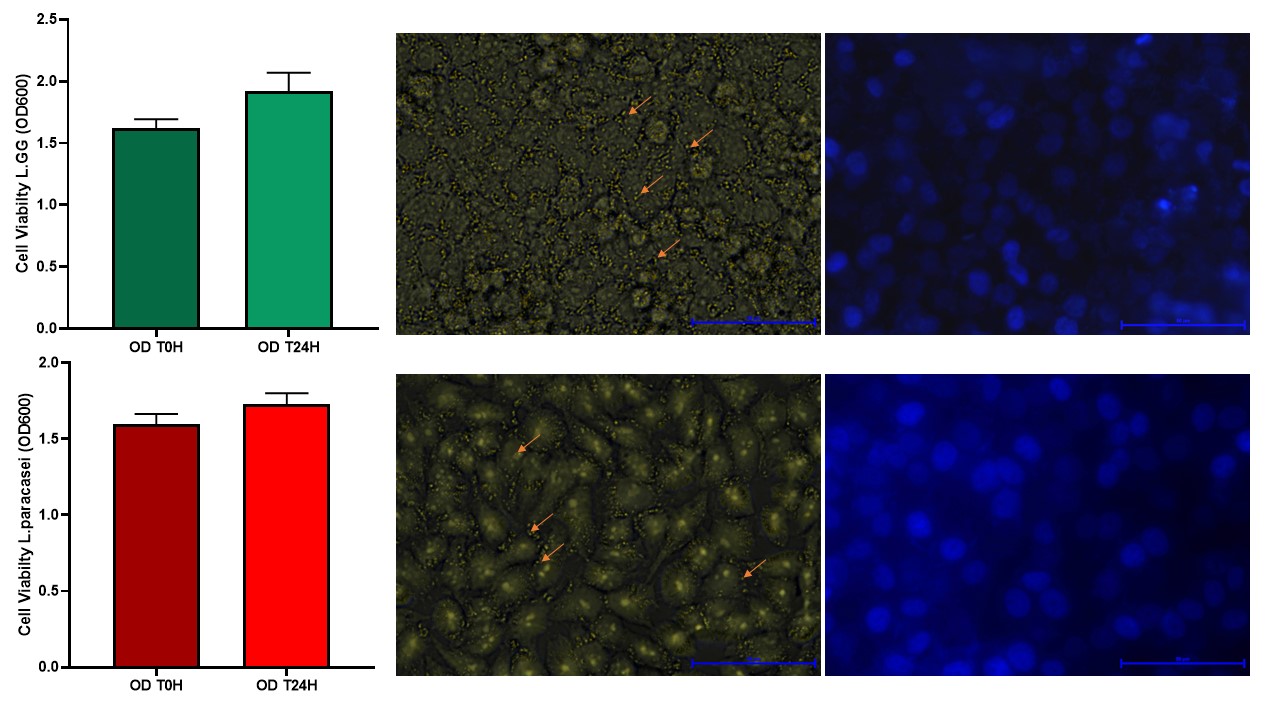

Supplement: Supplementary file 1 [file ijms-26-11148-s001.zip › Fig S.1.jpg]
